# Molecular identification and expression of sesquiterpene pathway genes responsible for patchoulol biosynthesis and regulation in *Pogostemon cablin*

**DOI:** 10.1186/s40529-019-0259-9

**Published:** 2019-07-02

**Authors:** Yun Tang, Liting Zhong, Xiaobing Wang, Hai Zheng, Likai Chen

**Affiliations:** 1 Research Center of Chinese Herbal Resource Science and Engineering, Key Laboratory of Chinese Medicinal Resource From Lingnan (Guangzhou University of Chinese Medicine), Ministry of Education, Joint Laboratory of National Engineering Research Center for the Pharmaceutics of Traditional Chinese Medicines, Guangzhou University of Chinese Medicine, Guangzhou, 510006 People’s Republic of China; 2 Guangdong Institute of Traditional Chinese Medicine, Guangzhou, 510520 People’s Republic of China

**Keywords:** *Pogostemon cablin*, Patchoulol, Terpenoid biosynthesis, Sesquiterpene, Pathway

## Abstract

**Background:**

Many commercially important drug and flavor compounds are secondary metabolites of terpenoid origin. *Pogostemon cablin*, a commercially important industrial and medicinal crop, accumulates abundant patchouli oil comprised of more than 24 unique sesquiterpene compounds, with the most abundant being patchouli alcohol.

**Results:**

In this study, we analyzed the *P. cablin* transcriptome library, obtaining 74 terpenoid biosynthesis-related genes, and identified their expression patterns in leaves, stems, and flowers. These genes are members of 15 different families, and we detected all the enzymes involved in the sesquiterpenes pathway that are responsible for patchoulol biosynthesis. Sequence structure, homology, conserved domain properties, and phylogeny of certain identified genes were systematically investigated. Color complementation assay was used to verify the functional activity of the MEP pathway proteins. Exogenous hormone treatment revealed that patchoulol synthesis is induced by methyl jasmonate (MeJA). Quantitative reverse-transcription PCR analysis indicated that the MVA pathway genes (acetoacetyl-CoA thiolase, 3-hydroxy-3-methylglutaryl-coenzyme A reductase, mevalonate diphosphate decarboxylase, and farnesyl diphosphate synthase) participate in patchoulol biosynthesis and are mediated by MeJA.

**Conclusions:**

Taken together, this is the first report of integrated analysis of *P. cablin* MVA and MEP pathway related genes, providing a better understanding of terpenoid and/or patchoulol biosynthesis in *P. cablin,* and the basis for improving patchoulol production through genetic engineering.

**Electronic supplementary material:**

The online version of this article (10.1186/s40529-019-0259-9) contains supplementary material, which is available to authorized users.

## Background

As specialized metabolites, plant terpenoids are of economic interest for drugs, nutraceuticals, flavors, fragrances, pigments, agrochemicals, and disinfectants. In the last three decades, the molecular biology, chemistry, and transcriptomics of terpenoid biosynthesis have attracted extensive interest (Bohlmann and Keeling 2008; Vranova et al. 2013; Wong et al. 2017). Considering the vital function of terpenoids in plant growth and the potentially significant value of metabolic engineering of biosynthesis pathways, genes encoding enzymes that participate in terpenoid biosynthesis have been identified and characterized in various medicinal plants, such as *Salvia miltiorrhiza* and *Artemisia annua* (Brown 2010; Ma et al. 2012). However, many of the genes encoding enzymes have not been identified because of the complexity of the terpene biosynthesis pathways.

All terpenoids are derived from the common precursors isopentenyl diphosphate (IPP) and dimethylallyl diphosphate (DMAPP), which are synthesized via two independent pathways: the cytoplasmic mevalonate (MVA) pathway and the plastidic methylerythritol 4-phosphate (MEP) pathway (Enfissi et al. 2005). Ordinarily, the MVA pathway supplies the precursors to sesquiterpenes, whereas the MEP pathway yields hemiterpenes, monoterpenes, and diterpenes. In the MVA pathway, six enzymes participate in a continuous catalytic formation of IPP including acetoacetyl-CoA thiolase (AACT, EC 2.3.1.9); hydroxy-3-methylglutaryl-CoA synthase (HMGS, EC 2.3.3.10); 3-hydroxy-3-methylglutaryl-CoA reductase (HMGR, EC 1.1.1.34); mevalonate kinase (MVK, EC 2.7.1.36); phosphomevalonate kinase (PMK, EC 2.7.4.2), and mevalonate diphosphate decarboxylase (MVD, EC 4.1.1.33) (Newman and Chappell 1999). Next, IPP and dimethylallyl pyrophosphate (DMAPP) are condensed from farnesyl diphosphate synthase (FPPS, EC 2.5.1.10) to form the sesquiterpene intermediate. The MEP pathway starts with the condensation of pyruvate with the C1 aldehyde group of d-glyceraldehyde 3-phosphate (GA-3P). The condensation reaction is catalyzed by 1-deoxy-d-xylulose-5-phosphate synthase (DXS, EC 2.2.1.7), and then the reaction is subsequently catalyzed by 1-deoxy-d-xylulose-5-phosphate reductoisomerase (DXR, EC 1.1.1.267), 2-C-methyl-d-erythritol-4-phosphate cytidylyltransferase (MCT, EC 2.7.7.60), 4-diphosphocytidyl-2-C-methyl-d-erythritol Kinase (CMK, EC 2.7.1.148), 2-C-methyl-d-erythritol 2,4-cyclodiphosphate synthase (MDS, EC 4.6.1.12), 4-hydroxy-3-methylbut-2-en-1-yl diphosphate synthase (HDS, EC 1.17.7.1), and 4-hydroxy-3-methylbut2-en-1-yl diphosphate reductase (HDR, EC 1.17.1.2) to form IPP (Lichtenthaler et al. 1997).

*Pogostemon cablin* (Blanco) Benth. (Lamiaceae) is an important medicinal and spice plant that contains at least 140 biologically active compounds, including terpenoids and flavonoids (Mallappa Kumara et al. 2015). Patchouli oil, which has many pharmacological uses, is produced from the dried stems and leaves of *P. cablin* (Lehui et al. 2018), and is used to relieve depression and stress, calm nerves, control appetite, and improve sexual desire; it also has insecticidal, antibacterial, and antifungal properties (Albuquerque et al. 2013; Rocha et al. 2018). As the main component of the essential oils produced by *P. cablin*, patchouli oil is rich in sesquiterpenes and the principal component is patchoulol, a tricyclic sesquiterpene widely used in perfumery goods and cosmetics (Paul et al. 2010; Sugimura et al. 2005). Patchoulol is also used as the starting compound in the chemical synthesis of the chemotherapeutic drug paclitaxel (Blowman et al. 2018; Holton et al. 1994; Immethun et al. 2013). However, the molecular characteristics of the enzymes involved in patchoulol biosynthesis are still unclear, and the exact molecular regulatory mechanism has not been reported.

In this paper, MVA and MEP pathway-related genes were isolated from the *P. cablin* transcriptome, and then the gene expression in different organs was analyzed. Sequence structure, homology, conserved domain characteristics, and functional verification of these genes were systematically studied. The content of patchoulol induced by exogenous hormones was also detected, and the dynamic expression of sesquiterpene pathway genes responding to MeJA was revealed, providing new insights into the sesquiterpene pathway genes responsible for patchoulol biosynthesis and regulation in *P. cablin.*

## Methods

### Plant materials

Branches of *P. cablin* were cutted from the plant grown in Yangjiang, Guangdong, China, North latitude 21°28′45″, longitude 111°16′35, and the technique of cottage was used to get more cutting seedlings. Seedlings were transplanted into flower pots at germination stage. 60-day-old for each sample were used in this study. The tissue-culture condition as follow: 25 ± 2  °C, relative humidity of 70%, illumination intensity of 130 μmol m^−2^ s^−1^ and photoperiod of 16-h-light/8-h-dark. Leaf, stem and flower tissues were from the same specimen. MeJA (Sigma, USA) from the solution containing 0.1% Tween-80 and 5% alcohol was sprayed onto the leaves of the plants to give the final concentration to 300 μM. Control group was sprayed with 0.1% alcohol solution. All samples were frozen and stored at − 80 °C until use.

### RNA and gene isolation

Total RNA was isolated from frozen tissues using the plant total RNA purification kit (GeneMark, China). RNA quantity was decided using a NanoPhotometer Ultra-micro spectrophotometer (Implen, Germany). High-quality RNAs were further used for gene cloning or calculation of gene expression. Sequences of terpenoid-related genes were retrieved from a *P. cablin* EST database, which was produced on an Illumina^®^ Hiseq platform (Illumina Inc., San Diego, CA, USA) (Chen et al. unpublished data). These terpenoid backbone enzymes contain *PatAACT*, *PatHMGS*, *PatHMGR*, *PatMVK*, *PatPMK*, *PatMVD*, *PatIPPI*, *PatDXS*, *PatDXR*, *PatMCT*, *PatCMK*, *PatMDS*, *PatHDS*, *PatHDR,* and *PatFPPS*. The primers were designed pursuant to unigenes that were retrieved using Primer3Plus (http://www.bioinformatics.nl/cgi-bin/primer3plus/primer3plus.cgi/) to magnify the open reading frames (ORFs) genes (Additional file 1: Table S1). First single-strand cDNA was synthesized using TransScript^®^ Reverse Transcriptase kit (TransGen, China), according to the manufacturer’s protocol. Polymerase chain reaction (PCR) was performed under the conditions according to the manufacturer’s instructions. The PCR products were then transformed into *E. coli* DH5α cells and the positive clone was isolated and sequenced.

### Sequence feature analysis

The theoretical pIs and Mws were predicted with the Compute pI/MW Tool r (http://web.expasy.org/compute_pi/) (Bjellqvist et al. 1994). The localizations of deduced proteins were performed using the BaCelLo server (http://gpcr2.biocomp.unibo.it/bacello/pred.htm) (Pierleoni et al. 2006). Transmembrane region was predicted using the TRMHMM server v 2.0 (http://www.cbs.dtu.dk/services/TMHMM-2.0/) (Krogh et al. 2001). Conserved domain and signal peptide were analyzed with the Pfam protein families d atabase (http://pfam.xfam.org/) (Finn et al. 2008). Multiple sequence alignment was carried out using DNAMAN program. Database searches for similar sequences were executed using the NCBIs BLAST network service. The phylogenetic analyses were using the Phylogeny.fr server (http://www.phylogeny.fr) (Dereeper et al. 2008).

### Analysis of volatile compounds in leaves

Mature leaves (0.2 g) were ground frizzed in liquid nitrogen, with 1.5 mL hexane ultrasonic extraction for 25 min, and then under the 56 °C water bath for 1 h. The sample was centrifuged at 8000 rpm for 10 min, and the supernatants were transferred into new vials for GC–MS analysis. GC–MS was performed using an Agilent 7890B Gas Chromatograph with 5977A inert Mass Selective Detector (Agilent, United States). The gas chromatograph was equipped with an HP-5MS capillary column (30 m × 250 mm × 0.25 mm film thickness). The oven temperature was programmed from 35 °C (5 min hold) to 300 °C at a rate of 12 °C/min. NIST14/Wiley275 Mass Spectral Library was used for metabolite identification. The terpene compounds were identified by the mass spectral library. The content of volatile terpenes was quantified by using cyclohexanone as an external standard.

### Quantitative reverse-transcription PCR (qRT-PCR)

For every sample, qRT-PCR was performed with the SYBR qPCR master mix (Vazyme) on CFX96 Real-Time PCR system (Bio-Rad, USA). Gene-specific primers were designed with NCBI and were described in Additional file 1: Table S1. The house-keeping of *P. cablin* served as an internal control. To calculate the relative gene expression levels, the 2^−∆∆t^ method was used (Livak and Schmittgen 2001). All samples had three biological replicates and two technical replicates.

### Color complementation assay of MEP-related and IPPI genes

The bacterial color complementation trial was used to identify the function of DXR, MCT and DXS by using pAC-BETA and pTrc-Atipi (Lange et al. 2000; Tong et al. 2015). The β-carotene biosynthetic pAC-BETA was brought into XL1-Blue to rebuild the synthesis pathway of β-carotene (Cunningham et al. 1996). Plasmid pAC-BETA contains a chloramphenicol resistance gene and four functional genes necessary for β-carotene biosynthesis, crtE, crtB, crtL, and crtY. Plasmid pTrc-Atipi contains an IPI gene and an ampicillin from *Arabidopsis thaliana*. pTrc-Atipi was digested with *Pst*I to remove the *Arabidopsis* IPI gene, and the modified plasmid was named pTrc. The MVA- and MEP-related genes (DXS, DXR, MCT, CMK, MDS, HDS, HDR, and IPPI) was amplified and then ligated into pTrc through *PstI* restriction enzyme to create recombinant expression plasmids. pTrc, pTrc-AtIPI and pTrc- MVA- and MEP-related genes were respectively transformed into XL1-Blue harboring pAC-BETA and the putative transformants were identified by selection medium. Finally, the 3 types of XL1-Blue were grown on the medium, containing ampicillin (150 μg/ml) and chloromycetin (50 μg/ml) to observe the color of the transformants after 48 h at 30 °C.

## Results

### Global identification and tissue expression analysis of novel transcripts involved in sesquiterpene biosynthesis in *P. cablin*

**Fig. 1:**
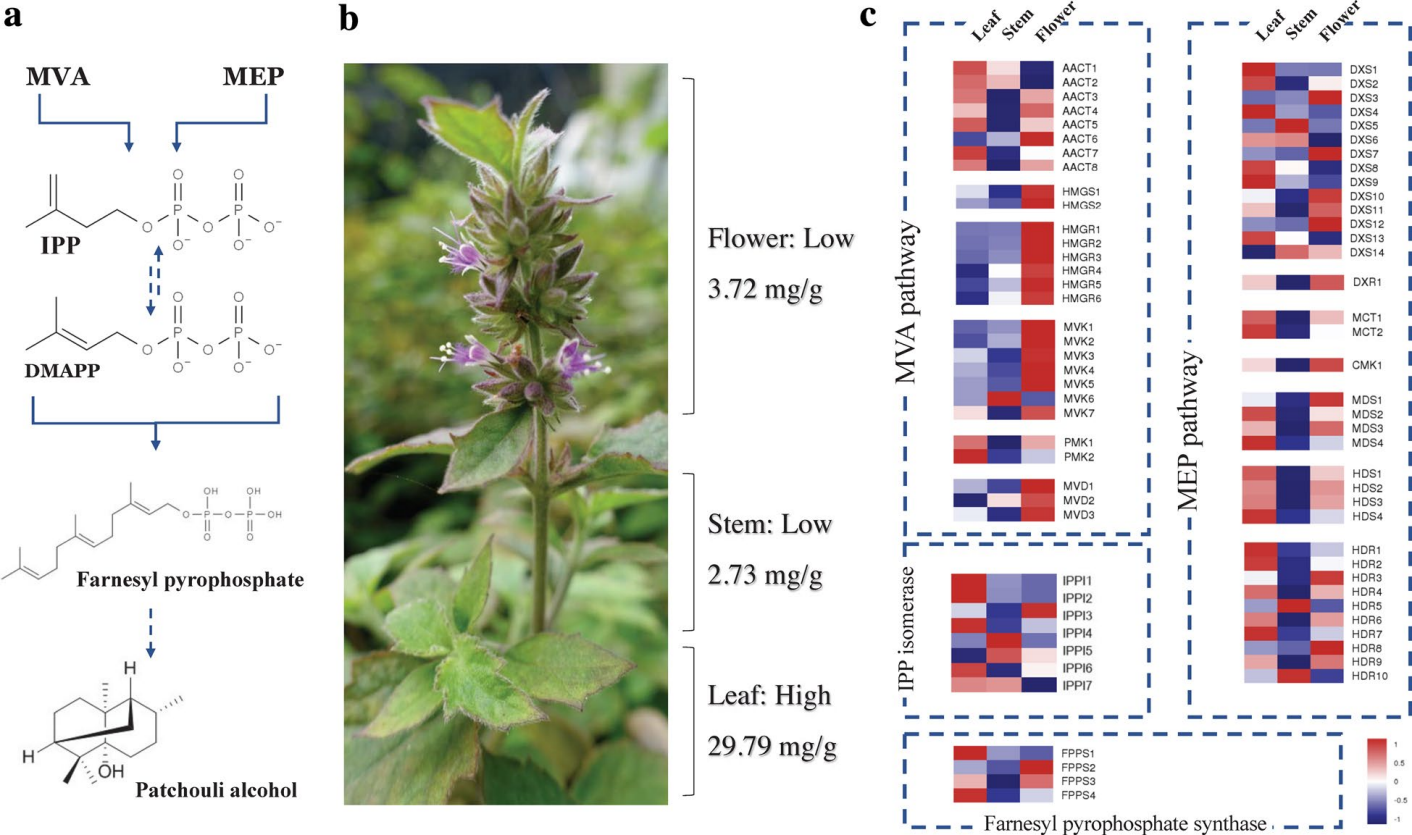
The major phytochemical structures and predicted terpenoid backbone pathway in *Pogostemon cablin* (Blanco) Benth. **a** The biosynthetic pathway of patchoulol in *P. cablin*. **b** Organelle specific biosynthesis of patchoulol in leaf, stem and flower of *P. cablin* plants, and their patchoulol contents were showed. **c** Expression patterns of sesquiterpene biosynthesis-related genes in various tissues of *P. cablin* plants. For heat map generated by OmicsShare, the color in each row represented the change of gene expression in leaf, stem, and flower. As shown in color key, navy or firebrick blocks indicate that the expression level of genes was downregulated or upregulated, respectively

In *P. cablin*, biosynthesis of sesquiterpene patchoulol is organelle specific, which exhibited significant highest content in leaves, but much lower in stems and flowers (Fig. 1b). To identify the genes, and their spatiotemporal expression, involved in *P. cablin* sesquiterpene biosynthesis, the transcript features and expressive abundance of 74 novel sesquiterpene biosynthesis-related genes were investigated, based on our transcriptome data that is summarized in Additional file 2: Table S2. These genes belong to 15 families, which encode all the enzymes that participate in the biosynthesis of the fundamental isoprene precursor IPP and its isomer DMAPP via the two distinct MVA and MEP pathways, and farnesyl diphosphate synthase, which is responsible for the biosynthesis of the 15-carbon precursor FPP (Fig. 1a). The sequence length of these 74 transcripts ranged from 804 to 3343 bp, with a mean mRNA length of 1972 bp. Most of the transcripts were well-annotated by the Nr, KEGG, GO, and SwissProt databases (Additional file 2: Table S2). We found that MVA-related genes exhibit the highest expression levels in flowers, whereas MEP-related genes exhibited the highest expression in leaves, and the expression of MVA and MEP pathway-related genes exhibit the lowest expression levels in stems. These results indicate diverse terpenoid metabolic activity levels in various tissues (Fig. 1c). Considering the importance of the MVA and MEP pathways in *P. cablin* patchoulol biosynthesis, understanding the molecular basis of these synthase genes are of importance.

### Identification of MVA-related genes in *P. cablin*

Precursors for cytosolic and mitochondrial isoprenoids are synthesized by the MVA pathway. Six enzymes participate in the MVA pathway (Fig. 1c), and a total of 26 transcripts were identified, including *PatAACCT1*-*8*, *PatHMGS1*-*2*, *PatHMGR1*-*6*, *PatMVK1*-*7*, *PatPMK1*-*2*, and *PatMVD1*-*3*. The initial reaction is catalyzed by AACT (EC 2.3.1.9), which condenses two molecules of acetyl-CoA to acetoacetyl-CoA. Eight AACT transcripts (*PatAACT1*–*PatAACT8*) were identified in the *P. cablin* transcriptome (Additional file 2: Table S2). Gene expression profiling shows that *PatAACT1*–*PatAACT8* are expressed in all tissues, with the highest expression being seen in leaves (Fig. 1). The expression of *PatAACT6* was the lowest in leaves, possibly because it may lack a complete conserved thiolase domain (Additional file 3: Fig. S1). *PatAACT1* and *PatAACT2* have very high sequence similarity and show similar expression levels, suggesting that they are repeating genes in *P. cablin*.

Hydroxymethylglutaryl-CoA synthase catalyzes the condensation reaction of acetyl-CoA and acetoacetyl-CoA to produce 3-hydroxy-3-methylglutaryl-CoA (HMGCoA). Two HMGS genes (*PatHMGS1* and *PatHMGS2*) were identified with a conserved hydroxymethylglutaryl-coenzyme A synthase domain in both the C-terminus and N-terminus (Additional file 3: Fig. S1). According to the prediction of BaCelLo, both *PatHMGS1* and *PatHMGS2* are localized to the nucleus. Both *PatHMGS1* and *PatHMGS2* are highly expressed in flowers, followed by leaves, but their lowest expression levels are seen in stems (Fig. 1c).

HMGR catalyzes the conversion of HMG-CoA to mevalonate acid, which is the first step in the biosynthesis of isoprenoid. Consistent with many other plant HMGRs, all of the six deduced *PatHMGR* proteins (*PatHMGR1*–*PatHMGR6*) contain two potential N-linked glycosylation sites (N-X-S/T), two HMG-CoA-binding motifs (EMPVGYVQIP and TTEGCLA), and two NADPH-binding motifs (DAMGMNM and GTCGGG) in the conserved C-terminal catalytic domain (Additional file 3: Fig. S1). However, *PatHMGR2* is missing a potential N-linked glycosylation site and two HMG-CoA-binding motifs (Additional file 3: Fig. S1). *PatHMGRs* are expressed mainly in flowers, followed by stems and leaves.

MVA is phosphorylated to the isopentenyl pyrophosphate precursor of the terpenoid in three successive reactions catalyzed by MVK, PMK, and MVD. *PatMVK* and *PatMVD* show the highest expression levels in flowers, followed by leaves, and stems. *PatMVK* and *PatMVD* contain the PTS2 peroxisomal targeting signal motif (Additional file 3: Fig. S1) previously found in MVK and PMK in other plant species, such as *Catharanthus roseus* (Simkin et al. 2011).

### Identification of MEP-related genes in *P. cablin*

The MEP pathway, which primarily exists in eubacteria and plants, produces IPP and DMAPP in plastids, and cross-flow of the prenyl-PP precursors between the cytosol and the plastid is likely to inherently regulate the accumulation of sesquiterpene. We detected 36 transcripts encoding enzymes that participated in the MEP pathway, including fourteen *DXS* genes, one *DXR* gene, two *MCT* genes, one *CMK* gene, four *MDS* genes, four *HDS* genes, and ten *HDR* genes; all seven MEP pathway enzymes were encoded.

*DXS* is the foremost enzyme of the MEP pathway, catalyzing the transketolase-type condensation reaction of glyceraldehyde-3-phosphate and pyruvate to produce 1-deoxy-d-xylulose-5-phosphate (DXP). All 14 *DXS* genes encode proteins with conserved domains and motifs in previously known DXSs (Additional file 3: Fig. S1), including the common thiamine pyrophosphatase-binding motif (except *PatDXS12*) and the pyridine binding DRAG domain, indicating that all *PatDXSs* have the same type of biochemical activity (Additional file 3: Fig. S1). The expression levels of *PatDXSs* in different tissues are diverse (Fig. 1), which is consistent with the *SmDXS* genes observed in *Salvia miltiorrhiza* (Ma et al. 2012). In plants, the DXS multigene family consists of three independent classes by functional partition. One of the independent classes specifically participates in the synthesis of essential terpenoids, such as photosynthetic pigments, and another synthesizes the secondary metabolites of specific terpenoids (Tong et al. 2015); the last is involved in the biosynthesis of isoprenoids like phytohormones that are necessitated at low levels. Among the fourteen *PatDXS* genes, *PatDXS1, PatDXS2, PatDXS8, PatDX9,* and *PatDXS13* belong to clade I, *PatDXS3*–*PatDXS7* are members of clade II, and *PatDXS10*, *PatDXS11, PatDXS12,* and *PatDXS14* belong to the more divergent clade III (Additional file 3: Figure S23). These results indicate the existence of members of all three DXS clades in *P. cablin* and indicate the different roles of each *PatDXS* gene in terpenoid biosynthesis.

The intramolecular rearrangement and reduction of DXP to MEP is catalyzed by DXR. Only one DXR gene was found (*PatDXR*), and the deduced PatDXR protein contains two NADPH binding motifs and two active sites of DXR (Additional file 3: Fig. S1), indicating that *PatDXR* has similar biological activity. In this study, *PatDXR* exhibited tissue-specific expression, with the highest expression levels being seen in the flowers, followed by leaves and stems (Fig. 1).

MEP is then converted to 4-(cytidine 5′-diphospho)-2-C-methyl-d-erythritol (CDP-ME) in a CTP-dependent reaction catalyzed by MCT. Two *PatMCT* genes were identified in our cDNA library, and the derived proteins contain the conserved IspD motifs, which are highly homologous with MCT of other plants (Additional file 3: Figs. S1, S10). Both *PatMCT1* and *PatMCT2* were detected to been highest expressed in leave tissues, and relatively weakly in flowers and stems (Fig. 1c). In the next three steps of the MEP pathway, the hydroxyl group in the C2 position of CME is further phosphorylated by CMK, and the resulting product 2-phospho-4-(cytidine 5′-diphospho)2-C-methyl-d-erythritol (PCME) is subsequently converted to 2-C-methyl-Derythritol 2,4-cyclodiphosphate (CMEC), which is then catalyzed by MDS. CMEC is then reduced by HDS to HMBPP. Based on our transcriptome data, we identified one *PatCMK* gene, four *PatMDS* genes, and four *PatHDS* genes (Additional file 3: Fig. S1). PatMDSs and PatHDSs are mainly expressed in leaves, and the expression patterns of PatMDSs and PatHDSs are comparable to that of other gene families in the MEP pathway.

HMBPP, produced under the catalysis of MDS, can be further converted into the isoprene precursor IPP by HDR, an enzyme that also plays a key role in the supply of plastidial terpenoid precursors (Botella-Pavia et al. 2004). In this research, ten *PatHDR* genes were confirmed in *P. cablin*, and their deduced proteins contain the conserved LyTB motif, which participates in the trunk line of the MEP pathway (Additional file 3: Fig. S1). The derived amino acid sequences of *PatHDR1* and *PatHDR7* show 89.68% homology. The two *PatHDR* genes have similar structures and are located in the same organelle, suggesting they are possibly derived from a gene replication event. Similarly, *PatHDR4* and *PatHDR9* show 88.89% homology and are located in chloroplasts, suggesting they may also be derived from a gene duplication event. *PatHDRs* show tissue-specific expression, which is higher in leaves than flowers and stems.

### Identification of IPPI and FPPS genes in *P. cablin*

Conversion of IPP to DMAPP and the equilibrium between IPP and DMAPP are controlled by IPPI with a reversible reaction (Berthelot et al. 2012). Eight IPPI genes (*PatIPPI1*–*PatIPPI8*) exist in the *P. cablin* transcriptome. Consistently, BaCelLo prediction suggests that *PatIPPI2* and *PatIPPI3* are cytoplasmic, whereas other PatIPPIs are localized in the nucleus. Furthermore, both *PatIPPI2* and *PatIPPI3* contain the PTS1 peroxisomal targeting signal motif (HKL) (Additional file 3: Fig. S11), suggesting they may target peroxisomes. *PatIPPI2* is mainly expressed in leaves, followed by stems and flowers. In contrast, *PatIPPI3* is mainly expressed in flowers. However, the levels of *PatIPPI3* are much lower than those of *PatIPPI2*, suggesting that *PatIPPI2* may participate in the synthesis of secondary terpenoids, such as patchoulol in *P. cablin*, while *PatIPPI3* probably participates in primary metabolism and has a housekeeping function.

FPPS catalyzes the sequential head-to-tail condensation of two molecules of IPP with one molecule of DMAPP to form the sesquiterpene precursor FPP. Four FPPS genes (*PatFPPS1*–*PatFPPS4*) were isolated and all of the deduced PatFPPS proteins contain five highly conserved domains, two of which are rich in the Asp motif (DDXXDD), considered to be IPP and DMAPP binding sites (Additional file 3: Fig. S1). PatFPPSs were expressed in all analyzed tissues, including leaves, stems, and flowers. Higher expression levels were found in leaves and flowers, indicating the key involvement of PatFPPS proteins in terpenoid biosynthesis in *P. cablin*.

### Molecular cloning and sequence analysis of the sesquiterpene biosynthesis-related genes

Based on the sequence feature and tissue expression pattern analysis, 15 genes were cloned and characterized from *P. cablin*; six MVA-related genes (Additional file 3: Figs. S2–S7) and seven MEP-related genes (Additional file 3: Figs. S8–S14), *PatIPPI* (Additional file 3: Fig. S15), and *PatFPPS* (Additional file 3: Fig. S16). The derived proteins exhibit different lengths, isoelectric points (pI), molecular weights, subcellular localizations, and transmembrane helix numbers (Table 1, Additional file 3: Figs. S2–S16). Multiple sequence alignment was conducted and a phylogenetic tree was constructed of these proteins (Additional file 3: Figs. S17–S31).

**Table 1:** Terpenoid biosynthesis-related genes (cloned) in *P. cablin*

Name	Pathway	Len^a^	pI	Mol.wt (kDa)	Loc^b^	TMH^c^	TPlen^d^
AACT	MVA	402	5.08	41.3	Cytoplasm	0	–
HMGS	MVA	460	6.61	50.7	Nucleus	0	–
HMGR	MVA	590	7.05	63.1	Cytoplasm	2	–
MVK	MVA	387	5.77	41.1	Cytoplasm	0	–
PMK	MVA	508	5.78	55	Nucleus	0	30
MVD	MVA	420	6.37	46.7	Nucleus	0	–
DXS	MEP	718	6.77	77.1	Chloroplast	0	56
DXR	MEP	473	5.93	51.7	Chloroplast	0	45
MCT	MEP	278	7.84	30.6	Chloroplast	0	21
CMK	MEP	210	4.64	23.2	Nucleus	0	–
MDS	MEP	193	6.13	20.5	Nucleus	0	13
HDS	MEP	742	6.1	82.6	Cytoplasm	0	–
HDR	MEP	457	5.61	51.4	Nucleus	0	33
IPPI		193	4.96	22	Nucleus	0	–
FPPS		349	5.63	40.1	Cytoplasm	0	–

^a^Len represents the number of amino acid residue

^b^Loc represents the protein localization predicted by BaCelLo

^c^TMH represents the number of predicted transmembrane helices

^d^TPlen represents the length of predicted presequence

### Functional complementation of MEP-related genes and *PatIPPI* activity in *E. coli*

**Fig. 2:**
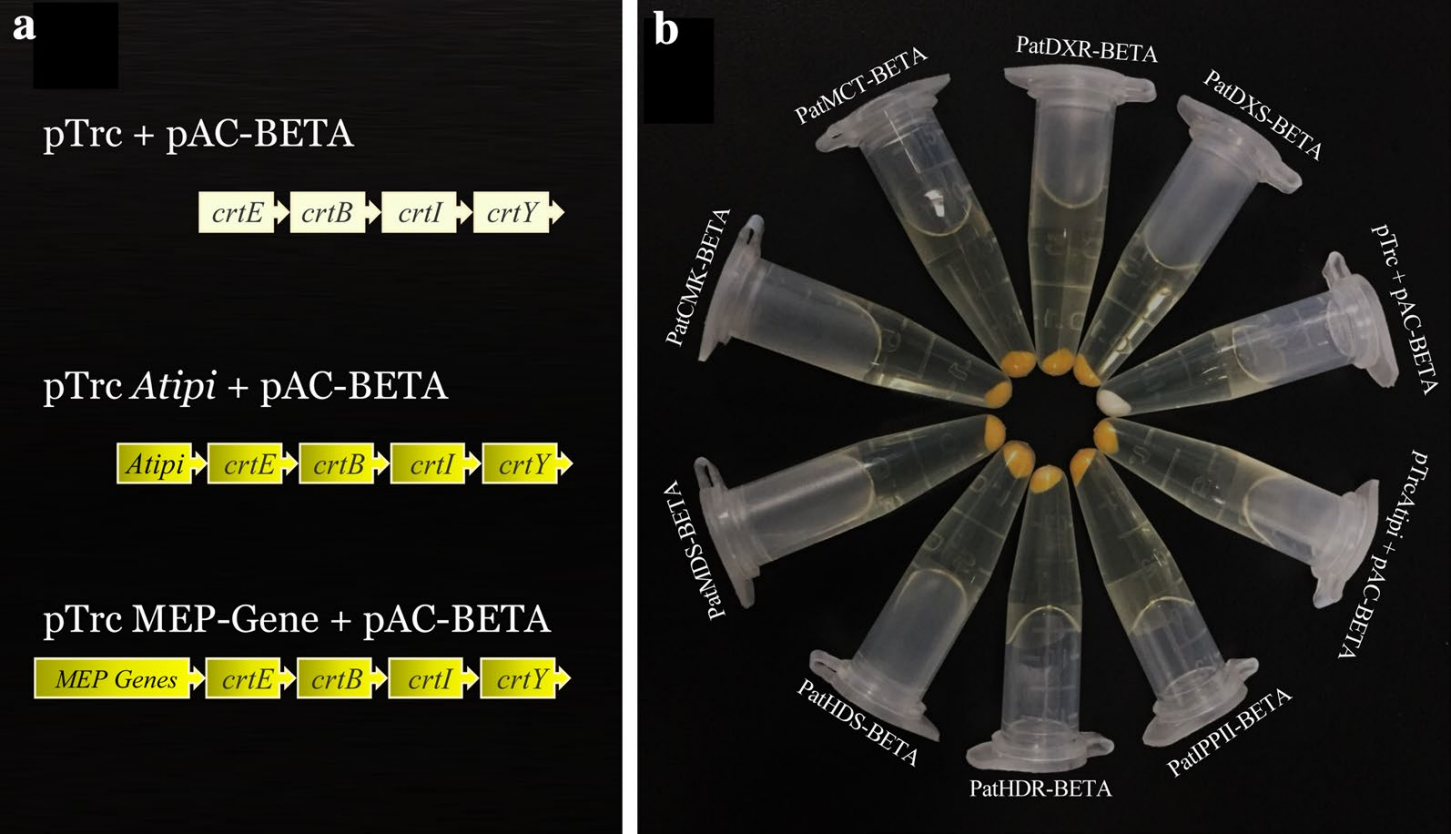
Functional complementation of MEP-related genes and *PatIPPI* activity, using *E. coil* strain XL1-Blue. **a** Illustration of the gene expression vectors used for functional complementation analysis in *E. coil*. **b** Visual observation of *E. coli* cultures consisting of pAC-BETA and complemented with plasmids containing target genes. In the selected medium, the bacterial clones of *E. coli* harboring pAC-BETA and pTrc were the raw color, while *E. coli* harboring pAC-BETA and pTrc-target genes were orange that was given by β-carotene

To verify the function of MEP-related genes, color complementary systems were used in *E. coli*. We constructed a bacterial expression vector of pTrc, pTrc-Pat (DXS, DXR, MCT, CMK, MDS, HDS, HDR, and IPPI), which was controlled by the pTrc promoter (a strong bacterial promoter). This was used to transform *E. coli* XL1-Blue competent cells with pAC-BETA to test the function (Fig. 2a). In the present study, the bacteria harboring pAC-BETA and pTrc formed natural colored colonies, whereas *E. coli* harboring both plasmids, pAC-BETA and pTrc-Pat (DXS, DXR, MCT, CMK, MDS, HDS, HDR, and IPPI), formed orange colonies, the color of which was derived from β-carotene (Fig. 2b).

### Biosynthesis of sesquiterpene patchoulol and expression of sesquiterpene genes were induced by MeJA

**Fig. 3:**
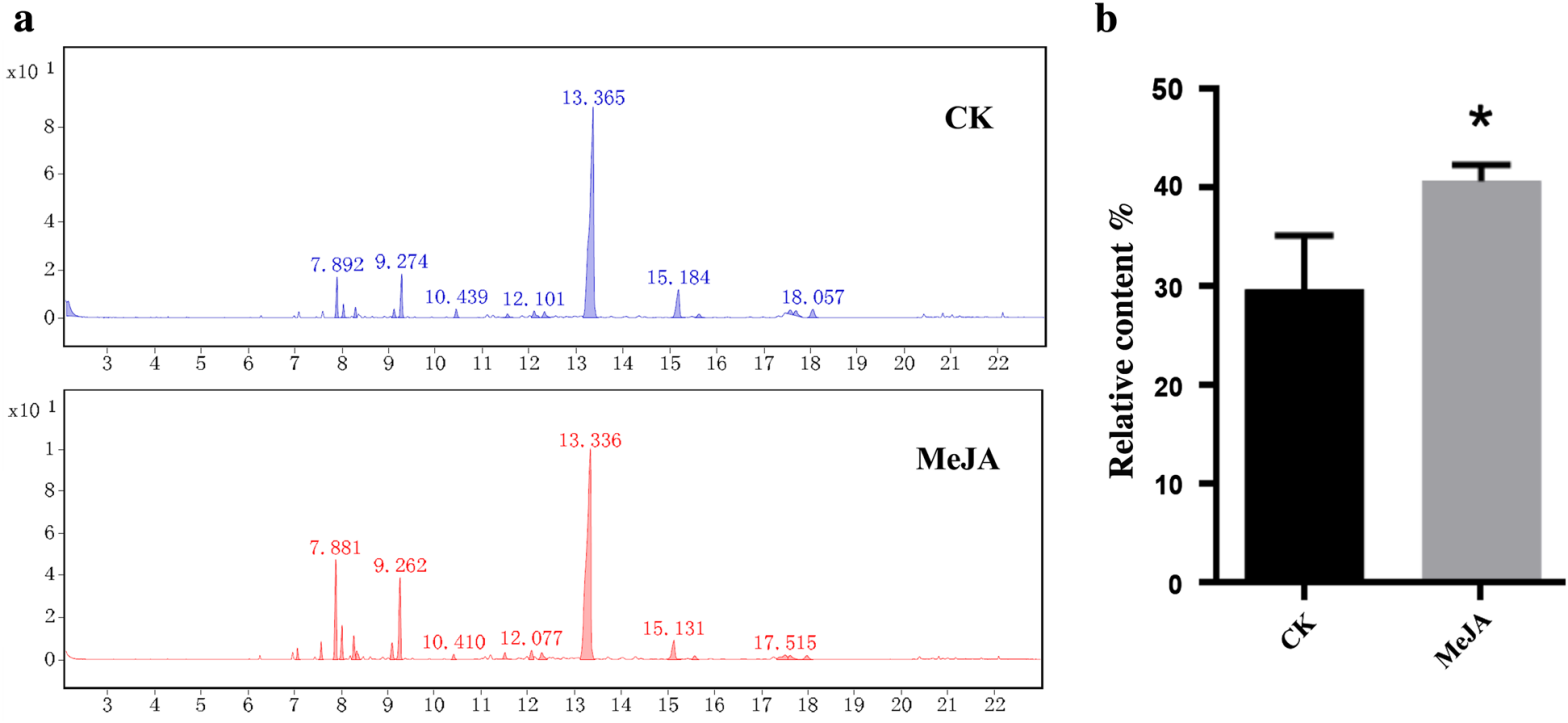
GC ± MS analysis of patchoulol in *P. cablin*. **a** Mass spectra at the retention time of patchoulol. Arrowheads indicate the retention time of patchoulol. **b** The accumulation of patchoulol in *P. cablin*. Student’s t-test was performed to identify significant differences. One asterisk (*) indicates a significant difference (0.01 < *P* < 0.05)

To identify the sesquiterpene biosynthetic pathways regulated by the elicitor, the content of patchoulol in leaves under ABA treatment, SA treatment, and MeJA treatment were measured. The results of the GC–MS showed that the content of patchoulol significantly increased by 37.03% after treatment with MeJA for 8 h (Fig. 3), and there were no significant changes under ABA or SA treatments. These results suggest that the role of MeJA as an effective abiotic elicitor accelerates the accumulation of patchoulol in *P. cablin* leaves and may be related to the expression of genes in the terpenoid biosynthetic pathway.

**Fig. 4:**
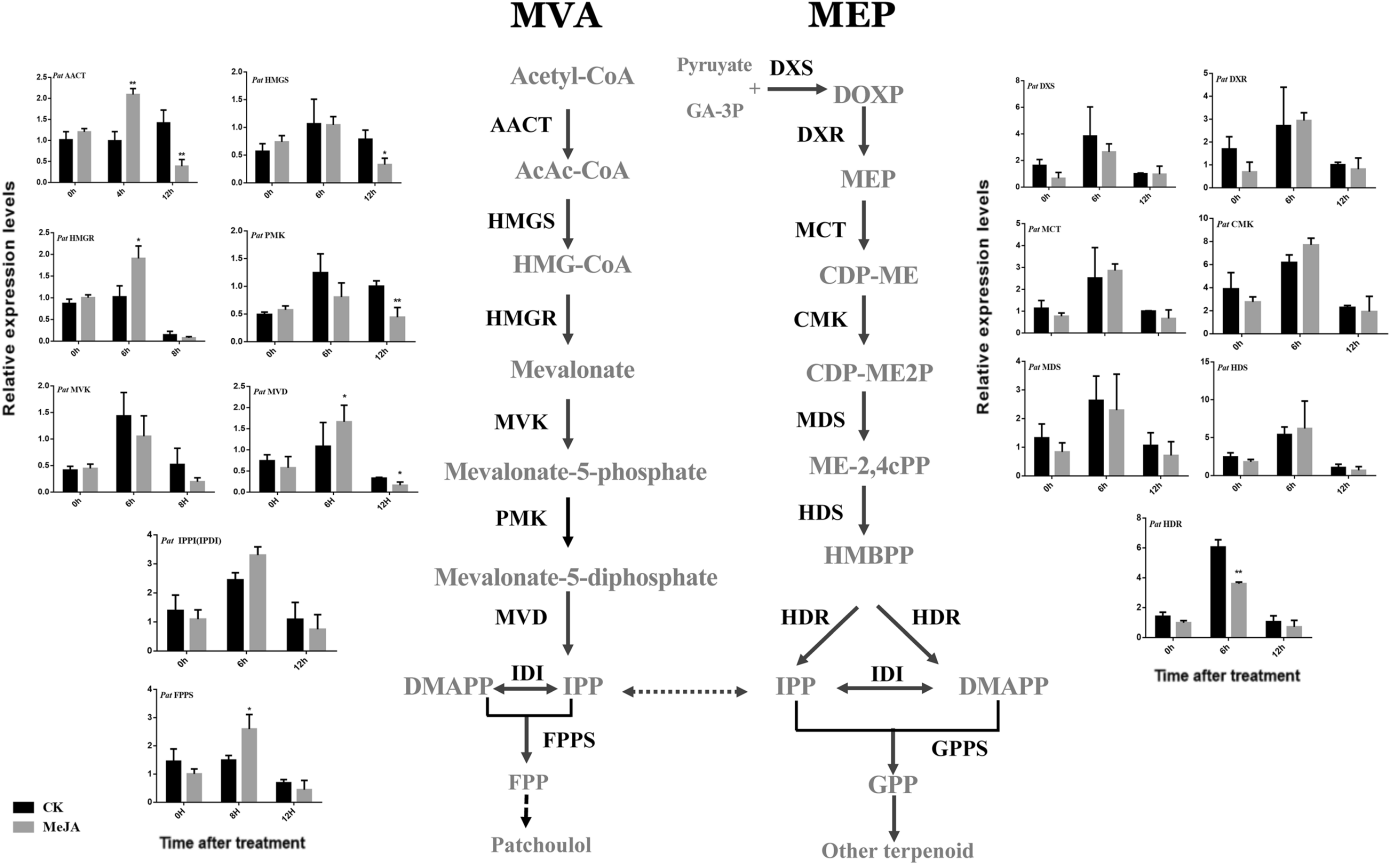
Study on the MeJA-induced expression patterns of sesquiterpene synthesis pathway genes. Graphs within the accumulation of patchoulol in *P. cablin* leaves treated with 300 μM MeJA, (symbols and their error bars represent means ± SD from three independent biological replicates). Student’s t-test was performed to identify significant differences. One asterisk (*) indicates a significant difference (0.01 < *P* < 0.05) and two asterisks (**) indicate a very significant difference (*P* < 0.01)

qRT-PCR was used to investigate the regulation pattern of sesquiterpene biosynthesis-related genes in leaves of *P. cablin* in response to MeJA. The results showed that the genes in the MVA pathway, including AACT, HMGR, and MVD genes, were dramatically up-regulated two to three-fold by MeJA. The expression levels peaked at 4–6 h post-treatment but then drastically decreased to levels slightly lower than the control at 24 h (Fig. 4). However, the MVA pathway genes HMGS, PMK, and MVK showed more gradual and mildly increased expression levels, possibly reaching the highest level of gene expression before or after 6 h. Expression of the MEP-related genes DXS, DXR, MCT, CMK, MDS, and HDS was changed slightly by MeJA induction. *PatHDR* was significantly down-regulated by MeJA at 6 h (Fig. 4). The downstream genes of patchoulol synthesis, including IPPI and FPPS genes, were elevated by MeJA treatment, although to varying degrees. In addition, the gene expression levels of the control changed with time, probably due to circadian response.

## Discussion

The growing number of sequenced omics data provides new perspectives in the research of functional genomics and the accuracy of annotating new genes for the illumination of biological processes. In this study, using a comprehensive approach, we isolated some terpenoid biosynthesis genes and described their sequence characteristics, expression abundance in different plant tissues, and the probable physiological functions of these genes. Tissue-specific expression patterns were found in members of the gene family, indicating they may play distinct roles in the biosynthesis of terpenoids. A total of 15 genes that likely participate in patchoulol synthesis have been cloned for further study. Phylogenetic analysis showed that these proteins had high similarity to those that participate in the biosynthesis of terpenoids in a number of plant species.

Regulation of MVA- and MEP-pathway genes occurs mainly at the transcriptional level, with both developmental and environmental cues as well as pathway feedback signals regulating gene expression (Hemmerlin et al. 2012). Their expression levels were elevated significantly and peaked 0–6 h after MeJA was applied, then quickly declined, returning to the control level at 12 h, thus representing a rapid, yet transient, response to MeJA (Fig. 4). *PatAACT*, *PatHMGR,* and *PatMVD* seem to play the main role in supplying the isoprene precursor for patchoulol biosynthesis. These results indicate that under MeJA treatment, the genes of the MVA pathway play an important regulatory role in the synthesis of patchoulol. This also illustrates the significance of performing systematic research on the genes encoding enzymes related to the MVA pathway.

The MEP pathway is mainly present in eubacteria and plants but is absent in other eukaryotes, including fungi and animals (Lange et al. 2000). In plants, enzymes involved in this pathway usually operate in plastids to synthesize monoterpenes, diterpenes, carotenoids, and the phytol chain of chlorophyll. *PatDXR*, *PatCMK,* and *PatHDS* genes in the MEP pathway may indirectly affect the supply of the IPP precursor. However, MEP-related genes showed lower levels of response to MeJA treatment at 12 h, probably because the response itself is slow. This suggests that the MEP pathway responded later to MeJA treatment than the MVA pathway. The color complementation assay showed that MEP-related genes in *P. cablin* can improve the accumulation of β-carotene, demonstrating that *PatDXS, PatDXR, PatMCT, PatCMK, PatMDS, PatHDS, PatHDR, and PatIPPI* had catalytic activity. *PatIPPI and PatFPPS* play significant roles in the second stage of patchoulol biosynthesis (Fig. 2).

*PatDXR*, *PatCMK,* and *PatHDS* of the MEP pathway may indirectly affect the supply of the IPP precursor. *PatIPPI and PatFPPS* play significant roles in the second stage of patchoulol biosynthesis. We found that downstream pathway genes (*PatFPPS*) were more influenced than upstream pathway genes (*PatAACT*, *PatHMGR,* and *PatMVD*), suggesting that *PatFPPS* is a critical enzyme of patchoulol synthesis. In this study, *PatFPPS* appeared to be the gene most activated by MeJA treatment (its expression increased an average of three-fold in treated leaves), which is consistent with the hypothesis that FPPS is the limiting enzyme of the sesquiterpene biosynthetic pathway (Bouvier et al. 2000; Brodersen et al. 2012; Buchanan et al. 2002; Budziszewski et al. 2001). The specific role of *PatFPPS* in sesquiterpene biosynthesis requires further study in order to better understand the pivotal position of FPPS in isoprenoid metabolism. These results are consistent with the role of MeJA as an effective abiogenic inducer, promoting the accumulation of patchoulol in *P. cablin* leaves, and point to probable co-regulation of the two pathways. In addition, no significant change of patchoulol content was found under ABA or SA treatments in our experiments, which was different from sesquiterpenoid, such as artemisinin (Lv et al. 2017). In *A. annua.*, ABA can activate transcription factor AaBZIP1, directly regulate the accumulation of artemisinin. SA can enhance the expression level of ADS and increases the AN, AA, and DHAA content, but the mechanism was unknown (Lv et al. 2017). Although both artemisinin and patchoulol are sesquiterpenoid, however, the transcriptional regulation mechanism may be very different, which mainly controlled by the transcriptional regulators and promoter sequence or motifs of synthetase genes. Furthermore, the different treatments of exogenous hormone may also lead to alterable effects on terpene metabolism.

## Conclusions

Globally, 74 terpenoid biosynthesis-related genes were identified in *P. cablin*, which were grouped into 15 families, including two single and 13 multigene families. The accumulation of patchoulol in treated samples, correlating with all the terpenoid biosynthesis genes analyzed, showed a significant increase in gene expression after MeJA treatment (Pei et al. 2018). Analysis of the expression of terpenoid biosynthesis genes confirmed how MeJA application also regulated the related biosynthetic pathway. The biosynthesis of patchoulol and the expression of sesquiterpene genes are principally controlled by the MVA pathway genes *PatAACT*, *PatHMGR*, *PatMVD*, and *PatFPPS* and was induced by MeJA. MEP-related genes and *PatIPPI* activity was confirmed through an *E. coli* functional complementation assay. These results provide a better understanding of terpenoid biosynthesis in *P. cablin* and other plant species and could provide the basis for improving patchoulol production through genetic engineering.

## Additional files

**Additional file 1: Table S1.** Primers used for clone and qRT-PCR.

**Additional file 2: Table S2.** Overview of 74 transcripts involved sesquiterpene biosynthesis in *P. cablin.*

**Additional file 3: Fig. S1.** Conserved domains (black boxes) of enzyme genes involved in terpenoid biosynthesis in *P. cablin.* **Fig. S2–S16.** cDNA and deduced protein sequences of the terpenoid biosynthesis-related genes (cloned) in *P. cablin.* **Fig. S17–S31.** Sequence alignment and phylogenetic analysis of deduced terpenoid biosynthesis-related proteins from *P. cablin* and various other plants.

## Data Availability

Sequence data generated in this study have been deposited in the NCBI Sequence Read Archive (SRA) database (http://www.ncbi.nlm.nih.gov/sra/) under the project number PRJNA511937 (accession numbers SRR8756845-SRR8756847, SRR8756845-SRR8756847, and SRR8756845-SRR8756847).

## Abbreviations

AACT: acetoacetyl-CoA thiolase
ABA: abscisic acid
CDP-ME: 4-(cytidine 5′-diphospho)-2-C-methyl-d-erythritol
CMEC: 2-C-methyl-Derythritol 2,4-cyclodiphosphate
CMK: 4-diphosphocytidyl-2-C-methyl-d-erythritol Kinase
DMAPP: dimethylallyl diphosphate
DXR: 1-deoxy-d-xylulose-5-phosphate reductoisomerase
DXS: 1-deoxy-d-xylulose-5-phosphate synthase
EST: expressed sequence tags
FPPS: farnesyl diphosphate synthase
GA-3P: d-glyceraldehyde-3-phosphate
GC–MS: gas chromatography–mass spectrometry
HDR: 4-hydroxy-3-methylbut2-en-1-yl diphosphate reductase
HDS: 4-hydroxy-3-methylbut-2-en-1-yl diphosphate synthase
HMBPP: (E)-4-hydroxy-3-methyl-but-2-enyl diphosphate
HMGCoA: 3-hydroxy-3-methylglutaryl-CoA
HMGR: 3-hydroxy-3-methylglutaryl-coenzyme A reductase
HMGS: hydroxy-3-methylglutaryl-coenzyme A synthase
IPP: isopentenyl diphosphate
IPPI: isopentenyl pyrophosphate isomerase
MCT: 2-C-methyl-d-erythritol-4-phosphate cytidylyltransferase
MDS: 2-C-methyl-d-erythritol 2,4-cyclodiphosphate synthase
MeJA: methyl jasmonate
MEP: methylerythritol-4-phosphate
MVA: mevalonate
MVD: mevalonate diphosphate decarboxylase
MVK: mevalonate kinase
Mw: molecular weight
ORFs: open reading frames
PCME: 2-phospho-4-(cytidine 5′-diphospho)2-C-methyl-d-erythritol
pI: isoelectric point
PMK: phosphomevalonate kinase
qRT-PCR: real-time polymerase chain reaction
SA: salicy acid
TMH: the number of predicted transmembrane helices
Tplen: represents the length of predicted presequence
