# Supplementary material for: Molecular identification and expression of sesquiterpene pathway genes responsible for patchoulol biosynthesis and regulation in Pogostemon cablin
Source: Bot Stud. 2019 Jul 2;60:11. doi: 10.1186/s40529-019-0259-9 (PMC6606680; doi:10.1186/s40529-019-0259-9)
Supplement: Supplementary file 3 — Additional file 3: Fig. S1. Conserved domains (black boxes) of enzyme genes involved in terpenoid biosynthesis in P. cablin. Fig. S2–S16. cDNA and deduced protein sequences of the terpenoid biosynthesis-related genes (cloned) in P. cablin. Fig. S17–S31. Sequence alignment and phylogenetic analysis of deduced terpenoid biosynthesis-related proteins from P. cablin and various other plants. [file 40529_2019_259_MOESM3_ESM.pdf]

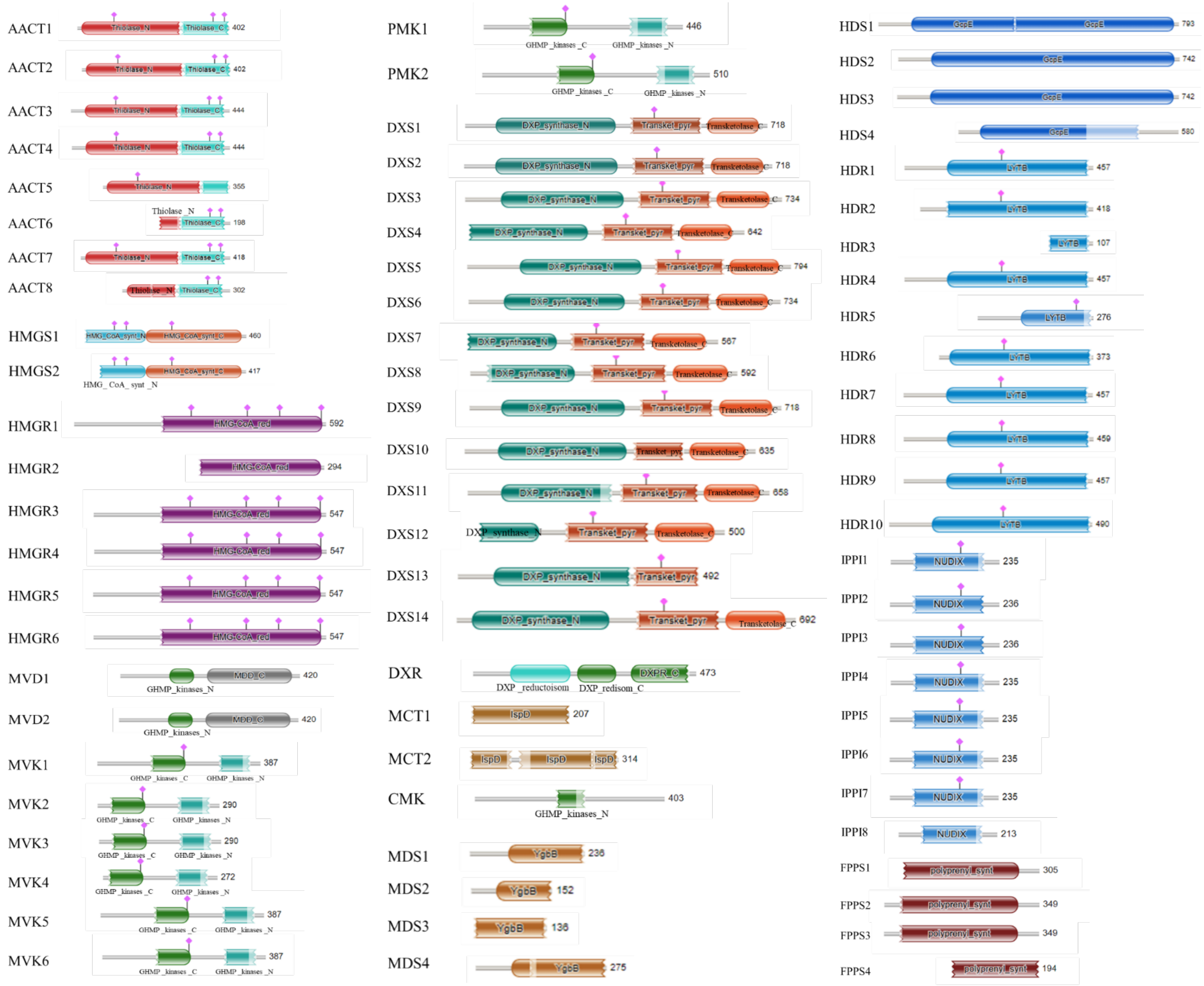

Figure S1. Conserved domains (colored boxes) of enzyme genes involved in terpenoid biosynthesis in *P. cablin*

```
1       ATGGCCGGAGGGGATTCAATTAAGCCACGAGATGTCTGTGTTGTCGGTGTTGCCCGAACACCTATGGGTGGTTTTCTCGGTTCACTTTCATCAGTATCTGCA
1        M  A  G  G  D  S  I  K  P  R  D  V  C  V  V  G  V  A  R  T  P  M  G  G  F  L  G  S  L  S  S  V  S  A
103     ACCAAACTTGGATCTATTGCTATTGAGAGTGCCCTAAAGAGAGCGAATATTGAACCAACACTTGTTCAAGAACTTTTCTTTGGAAATGTTCTCAGTGCAAAC
35       T  K  L  G  S  I  A  I  E  S  A  L  K  R  A  N  I  E  P  T  L  V  Q  E  L  F  F  G  N  V  L  S  A  N
205     TTAGGGCAAGCTCCAGCCAGGCAGGCAGCGATAGGTGCAGGTATCCCTAATACAGTGGTCTGTACAACTATCAACAAAGTCTGTGCCTCTGGAATGAAAGCA
69       L  G  Q  A  P  A  R  Q  A  A  I  G  A  G  I  P  N  T  V  V  C  T  T  I  N  K  V  C  A  S  G  M  K  A
307     ACTATATTAGCATCACAAACTATCCAGTTGGGAATCAATGATGTTGTAGTGGCTGGTGGTATGGAGAGCATGTCTAATGTCCCAAAGTACCTCGCAGAAGCA
103      T  I  L  A  S  Q  T  I  Q  L  G  I  N  D  V  V  V  A  G  G  M  E  S  M  S  N  V  P  K  Y  L  A  E  A
409     AGGAAAGGATCTCGACTTGGACACGATTCGCTGGTTGATGGAATGCTGAAAGATGGACTGTGGGATGTTTATAGTGATGTTGGAATGGGGGTCTGTGCTGAA
137      R  K  G  S  R  L  G  H  D  S  L  V  D  G  M  L  K  D  G  L  W  D  V  Y  S  D  V  G  M  G  V  C  A  E
511     TTATGTGCTGAGCATCATAGTATTACAAGGGAGCAGCAGGATGATTATGCAGTCCAAAGCTTTGAGCGTGGAATTGCTGCTCAAGATGCTGGCTCCTTTGCA
171      L  C  A  E  H  H  S  I  T  R  E  Q  Q  D  D  Y  A  V  Q  S  F  E  R  G  I  A  A  Q  D  A  G  S  F  A
613     TGGGAGATTGTTCCAGTTGAAGTGTCTGGGGGGAGAGGAAGACCCTCAACTATTGTTGATAAGGATGAAGGTCTTGGAAAGTTTGATGCTGCAAAGTTGAGG
205      W  E  I  V  P  V  E  V  S  G  G  R  G  R  P  S  T  I  V  D  K  D  E  G  L  G  K  F  D  A  A  K  L  R
715     AAGTTAAGACCAAGTTTCAAAGAAACTGGTGGTACTGTCACTGCTGGCAATGCTTCTAGTATAAGTGATGGTGCTGCTGCTCTTGTTTTAGTAAGTGGGCAG
239      K  L  R  P  S  F  K  E  T  G  G  T  V  T  A  G  N  A  S  S  I  S  D  G  A  A  A  L  V  L  V  S  G  Q
817     AAAGCTTTGGAGCTTGGGCTTAAAGTCATTGCAAAGATCTCAGGATATGCTGATGCTGCTCAAGCTCCGGAATTATTTACCACTGCCCCAGCTCTTGCAATT
273      K  A  L  E  L  G  L  K  V  I  A  K  I  S  G  Y  A  D  A  A  Q  A  P  E  L  F  T  T  A  P  A  L  A  I
919     CCAAAAGCAATTTCTAATGCTGGTTTAGAAGCATCTAAAATAGATTATTACGAAATCAATGAAGCCTTTGCGGTGGTGGCTCTTGCAAATCAGAAGCTACTG
307      P  K  A  I  S  N  A  G  L  E  A  S  K  I  D  Y  Y  E  I  N  E  A  F  A  V  V  A  L  A  N  Q  K  L  L
1021    GATCTTAGTCCTGAAAAAGTCAATGTACACGGTGGAGCTGTTTCGCTGGGGCATCCTCTTGGCTGCAGTGGTGCTCGTATCTTGGTCACACTTTTGGGGGTG
341      D  L  S  P  E  K  V  N  V  H  G  G  A  V  S  L  G  H  P  L  G  C  S  G  A  R  I  L  V  T  L  L  G  V
1123    CTGAGACAGAAGAACAGAAAGTATGGAGTTGGTGGCGTTTGCAATGGGGGAGGAGGTGCCTCAGCGCTTGTGGTTGAGCTTGTGTGA
375      L  R  Q  K  N  R  K  Y  G  V  G  G  V  C  N  G  G  G  G  A  S  A  L  V  V  E  L  V  *
```

Figure S2. Nucleotide sequence and deduced amino acid sequence of the *PatAACT*.

1	ATGGCCAAAAATGTCGGAATCCTCGCCATGGAAATCTACTTTCCTCCAACTTGCATCCAGCAGGAAGTGCTGGAGGCTCATGACGGAGCAAGCAAAGGGAAA
1	M A K N V G I L A M E I Y F P P T C I Q Q E V L E A H D G A S K G K
103	TACACAATTGGCCTTGGCCAAGATTGCATGGCATTTTGTTCTGAGGTTGAAGATGTTATCTCCATGAGTATGACAGCGGTAACTTCACTCCTAGAAAAGTAT
35	Y T I G L G Q D C M A F C S E V E D V I S M S M T A V T S L L E K Y
205	GATGTTGATCCAAAGCATATTGGTCGCCTTGAAGTTGGAAGTGAGACTGTACTTGATAAAAGCAAATCAATTAAGACATTTTTAATGCCAATCTTTGAGAAA
69	D V D P K H I G R L E V G S E T V L D K S K S I K T F L M P I F E K
307	TGTGGAAATACTGACATCGAAGGTGTTGACTCAAGCAATGCGTGCTATGGTGGTACTGCAGCACTATTTAACTGTGTCAATTGGGTGGAAAGTAGTTCTTGG
103	C G N T D I E G V D S S N A C Y G G T A A L F N C V N W V E S S S W
409	GATGGAAGATATGGTCTTGTTGTCTGCACTGACAGTGCGGTCTATGCTGAGGGACCAGCTAGGCCAACTGGAGGGGCTGCTGCTATTGCGATGCTAATAGGA
137	D G R Y G L V V C T D S A V Y A E G P A R P T G G A A A I A M L I G
511	CCAAATGCTCCGATTGCTTTTGAAAGCAAGCTCAGGGCGAGTCACATGGCTCATGCTTATGATTTCTACAAGCCTGATCTTGCCAGTGAATATCCAGTTGTT
171	P N A P I A F E S K L R A S H M A H A Y D F Y K P D L A S E Y P V V
613	GATGGAAAGCTTTCTCAGACATGTTACCTTATGGCACTGGATTCTTGTTACGAAAGCTTATGTCAAAAGTATAAGAAGTTGGAGGGCAAGCAATTCTCAATC
205	D G K L S Q T C Y L M A L D S C Y E S L C Q K Y K K L E G K Q F S I
715	TCTGATGCTGACTACTTTGTATTTCATTCACCTTACAACAAGCTTGTACAGAAGAGCTTTTCTAGATTGTTATACAATGATTTCTTGAGGAAAGCCAGCTCA
239	S D A D Y F V F H S P Y N K L V Q K S F S R L L Y N D F L R K A S S
817	ATTGATGAGGCTGCTAAAGAAAAGCTGGCACCATTTTCATCACTAAGCATTGATGAAAGCTACCAAAGCCGTGATCTTGAGAAGGCATCCCAACAAGTTGCG
273	I D E A A K E K L A P F S S L S I D E S Y Q S R D L E K A S Q Q V A
919	AAACCATTCTATGATGCTAAGGTTCAGCCATCTACACTCATTCCGAAACAGGTGGGGAACATGTATACTGCATCACTCTATGCTGCATTTGCCTCCCTCATC
307	K P F Y D A K V Q P S T L I P K Q V G N M Y T A S L Y A A F A S L I
1021	CACAACAAAGCAAGCTCGCTGGCTGGACAACGGGTCGTATTATTTCCTATGGCAGTGGTCTGACATCCATCATGTTTCTCTTCATCTAAACAAGGGACAA
341	H N K A S S L A G Q R V V L F S Y G S G L T S I M F S L H L N K G Q
1123	CATCCTTTTACCTTGTCTAACATTGCCACCATCATGAATGTCTCGGCCAAGTTGAAGGCTCGACATGAGTTACCACCTGAAAAATTCGTCGAAATTATGAAG
375	H P F T L S N I A T I M N V S A K L K A R H E L P P E K F V E I M K
1225	TTGATGGAGCACAGATACGGAGGCAAAGACTTCATCACCAGCAAGGACTGTACTCTTCTACCACCAGGTTCATACTATCTCACTGAGGTGGATTCTAAATAC
409	L M E H R Y G G K D F I T S K D C T L L P P G S Y Y L T E V D S K Y
1327	AGAAGATTCTATGCGAAGAAGGCTGCTGAAAATGGCACTGTCGTGAATGGCCACTGA
443	R R F Y A K K A A E N G T V V N G H *

Figure S3. Nucleotide sequence and deduced amino acid sequence of the *PatHMGS*.

```
1       CTTCTTCCTGAAGAGGGCGAAGAATTTGATCCATAAATCGCAATTCACACCATGGCCGCCGCCGCCGCTTCTCACCGCCGACCAGTTAAGGACTCCGACCTT
1        L  L  P  E  E  G  E  E  F  D  P  *  I  A  I  H  T  M  A  A  A  A  A  S  H  R  R  P  V  K  D  S  D  L
103     CAGAAGATGCCGGCGAAGGGCGGCCACACTACCCTCTCCGACTCCGCCAAGCCCACCGCCACCGATGCTGTCCCCCTCCCGCTCTACATCACCAACACCGTC
35       Q  K  M  P  A  K  G  G  H  T  T  L  S  D  S  A  K  P  T  A  T  D  A  V  P  L  P  L  Y  I  T  N  T  V
205     TTCTTCACGCTCTTCTTCTCCGTCGTCTATTTCCTGCTCCTCCGGTGGCGGGAGAAGATCCGCAACTCCACCCCTCTCCACGTCGTTACCCTTTCCGAAATC
69       F  F  T  L  F  F  S  V  V  Y  F  L  L  L  R  W  R  E  K  I  R  N  S  T  P  L  H  V  V  T  L  S  E  I
307     TCCGCCATCGTCGTCTTCTTCGCCTCCTTCATCTACCTCCTTGGCTTCTTCGGCCTGGGCTTCGTCCAGTCGCTCATTATTCCCCGCTCTTCGCACGATGAG
103      S  A  I  V  V  F  F  A  S  F  I  Y  L  L  G  F  F  G  L  G  F  V  Q  S  L  I  I  P  R  S  S  H  D  E
409     ATTTTGGATGACGATGAAATTATTGAGGATTTCGATCAAATGATGCTTAAGGAAGACTCACGCGCCGCCCCCTGCGCCGCTGCTCCGCCAACTGACGCCTGT
137      I  L  D  D  D  E  I  I  E  D  F  D  Q  M  M  L  K  E  D  S  R  A  A  P  C  A  A  A  P  P  T  D  A  C
511     AAGATTTCTTCCCCCAAACCGATTAAGAAAATCGTTGATGATTTTTGCCGGTTGTTCCCCTCTCCCCCGAGGAGGACGAGGAGATCGTGAAGTCGGTTGTG
171      K  I  S  S  P  K  P  I  K  K  I  V  D  D  F  L  P  V  V  P  L  S  P  E  E  D  E  E  I  V  K  S  V  V
613     GAAGGGAAAATCCCCTCCTACGCCCTAGAATCGAAGTTGGGCGATTGCCGCCGCGCCGCTGCCATCCGCCGTGAGGCCCTGCAGCGCACCACCGGGAAATCC
205      E  G  K  I  P  S  Y  A  L  E  S  K  L  G  D  C  R  R  A  A  A  I  R  R  E  A  L  Q  R  T  T  G  K  S
715     CTAGACGGTTTGCCTCTGGAAGGATTCAATTACGAAGCTATACTAGGTCAGTGCTGCGAAATGCCCGTAGGTTACGTCCAGATTCCGGTGGGGATCGCCGGG
239      L  D  G  L  P  L  E  G  F  N  Y  E  A  I  L  G  Q  C  C  E  M  P  V  G  Y  V  Q  I  P  V  G  I  A  G
817     CCTCTGTTGATGAACGGCGTGGAGTATTCGGTTCCCATGGCGACTACAGAGGGGTGCCTTGTTGCCAGCACCAACAGGGGATGCAAGGCCATTTACGCTTCT
273      P  L  L  M  N  G  V  E  Y  S  V  P  M  A  T  T  E  G  C  L  V  A  S  T  N  R  G  C  K  A  I  Y  A  S
919     GGCGGAGCCACCAGCATCATCATGAAAGACGGCATGACGAGAGCGCCGGTTGTCAGATTCGGAACGGCGAAAAGAGCTGCGGAGTTGAAGTTCTTCCTGGAA
307      G  G  A  T  S  I  I  M  K  D  G  M  T  R  A  P  V  V  R  F  G  T  A  K  R  A  A  E  L  K  F  F  L  E
1021    AACCCTCTCAATTTCGAAAGCCTGTCACAGTTATTCAACAGCTCCAGCCGATTCGGAAGGCTCCAGAACATCAAATGCGCCGTCGCCGGAAAGAATCTGTAC
341      N  P  L  N  F  E  S  L  S  Q  L  F  N  S  S  S  R  F  G  R  L  Q  N  I  K  C  A  V  A  G  K  N  L  Y
1123    ATGAGGTTCTGCTGCACAACAGGGGACGCCATGGGCATGAACATGGTATCTAAGGGTGTCCAGAATGTTCTAGACTTCCTCAACAAAGAGTTTCCGGATATG
375      M  R  F  C  C  T  T  G  D  A  M  G  M  N  M  V  S  K  G  V  Q  N  V  L  D  F  L  N  K  E  F  P  D  M
1225    GATGTCATCGGCATCTCCGGCAACTACTGTTCGGATAAAAAACCAGCTGCAGTGAATTGGATTGAGGGAAGGGGGAAATCAGTAGTATGCGAGGCCACAATC
409      D  V  I  G  I  S  G  N  Y  C  S  D  K  K  P  A  A  V  N  W  I  E  G  R  G  K  S  V  V  C  E  A  T  I
1327    CCAGAAGAAGTGGTCAAGAAGGTTCTCAAAACCGACGTCGCTTCACTTGTCGAGCTCAACATGCTGAAGAACCTGACAGGTTCCGCCATGGCCGGAGCTCTG
443      P  E  E  V  V  K  K  V  L  K  T  D  V  A  S  L  V  E  L  N  M  L  K  N  L  T  G  S  A  M  A  G  A  L
1429    GGTGGGTTCAACGCACACGCCAGCAACATCGTCTCCGCCGTGTACATCGCAACAGGGCAGGACCCCGCACAGAACGTGGAGAGCTCCCACTGCATCACCATG
477      G  G  F  N  A  H  A  S  N  I  V  S  A  V  Y  I  A  T  G  Q  D  P  A  Q  N  V  E  S  S  H  C  I  T  M
1531    GTGGAAGCCGTCAACGGCGGAAAGGACCTCCACATCTCCGTGACGATGCCATCCATCGAGGTGGGCACAGTCGGCGGCGGCACCCAGCTGGCATCCCAGTCA
511      V  E  A  V  N  G  G  K  D  L  H  I  S  V  T  M  P  S  I  E  V  G  T  V  G  G  G  T  Q  L  A  S  Q  S
1633    GCCTGCCTGAACCTGTTGGGCGTGAAGGGCGCCAGCAAAGAGGTCCCCGGAGCAAACGCGAGGCTGCTGGCGACGATCGTAGCAGGGTCGGTTCTGGCGGGA
545      A  C  L  N  L  L  G  V  K  G  A  S  K  E  V  P  G  A  N  A  R  L  L  A  T  I  V  A  G  S  V  L  A  G
1735    GAGCTGTCTCTGATGTCGGCCATAGCAGCAGGGCAGCTGGTGAAGAGCCACATGAAGTACAACAGATCGACCAAGGATGTGCGCAAGTGAGGTATGTATGTA
579      E  L  S  L  M  S  A  I  A  A  G  Q  L  V  K  S  H  M  K  Y  N  R  S  T  K  D  V  R  K  *  G  M  Y  V
1837    GAGAGAGATCCGCCATTGATGGAGGTAGGGAGGATTGCAGAGTCCATGTACTTGTAGTTAAAAAGAATAATGGCTTGGTAATGGTAATGGTAATAGTAATAG
613      E  R  D  P  P  L  M  E  V  G  R  I  A  E  S  M  Y  L  *  L  K  R  I  M  A  W  *  W  *  W  *  *  *  *
1939    TAGTGTTGTGCACTCCATCGGAAACACACGACGGAGGTGACCCATTAATA
647      *  C  C  A  L  H  R  K  H  T  T  E  V  T  H  *
```

Figure S4. Nucleotide sequence and deduced amino acid sequence of the *PatHMGR*.

```
1       ATGGAGGTCAGAGCCAGAGCGCCCGGGAAAATCATACTCGCCGGCGAACATGCCGTTGTTCACGGATCGACCGCCGTTGCCGCAGCCATCGATCTCTATACT
1        M  E  V  R  A  R  A  P  G  K  I  I  L  A  G  E  H  A  V  V  H  G  S  T  A  V  A  A  A  I  D  L  Y  T
103     TACGTCTCTCTTCGATTCCCCACTCCAGAGGAGAATGATGATGCACTAAAACTCCACCTCAAGGATTTGGACTTAGAATTCTCTTGGCCAGTTGGAAAGATT
35       Y  V  S  L  R  F  P  T  P  E  E  N  D  D  A  L  K  L  H  L  K  D  L  D  L  E  F  S  W  P  V  G  K  I
205     AAGGAAGTTCTGCCTGACCAGGGCAACCATGCTGCTTCCTCTCCTATATCATGTTCGTTTGAGGCCACAAAAGCCATTGCTTCTTTAGTTGAAGAACTAAAT
69       K  E  V  L  P  D  Q  G  N  H  A  A  S  S  P  I  S  C  S  F  E  A  T  K  A  I  A  S  L  V  E  E  L  N
307     ATTCCAGAGGCTAAAATTGCACTTGCTTCGGGTGTTTCAGCTTTTCTTTGGCTTTATACTTCGATACATGGTTATAAACCAGCTAAAGCTGTTGTGACCTCT
103      I  P  E  A  K  I  A  L  A  S  G  V  S  A  F  L  W  L  Y  T  S  I  H  G  Y  K  P  A  K  A  V  V  T  S
409     GAGCTGCCATTAGGCTCTGGCTTGGGTTCTTCTGCTGCATTATGTGTCGCACTCTCTGCTGCCCTCCTTGCTTTGTCTGATTCTGTGAAATTGGATTTTAGC
137      E  L  P  L  G  S  G  L  G  S  S  A  A  L  C  V  A  L  S  A  A  L  L  A  L  S  D  S  V  K  L  D  F  S
511     CATCAAGGTTGGCAAGTGTTTGGAGACAGTGAGCTAGAACTGGTAAACAAGTGGGCCTATGAAGGTGAAAAGATGATTCATGGAAAGCCATCGGGGATTGAC
171      H  Q  G  W  Q  V  F  G  D  S  E  L  E  L  V  N  K  W  A  Y  E  G  E  K  M  I  H  G  K  P  S  G  I  D
613     AACACAGTAAGCACCTATGGGAACATGATAAAATTTAGGTCGGGTGAGCTGACACGTATCAAGACAAACATGCCTCTGAAAATGCTAATAAGTAACACAAAA
205      N  T  V  S  T  Y  G  N  M  I  K  F  R  S  G  E  L  T  R  I  K  T  N  M  P  L  K  M  L  I  S  N  T  K
715     GTTGGAAGAAATACAAAGGCATTGGTAGCTGGTGTATCAGAAAGGGCAATGAGACATCCCAGTGCCATGACTTCTGTATTCAATGCTGTTGATTCCATCAGC
239      V  G  R  N  T  K  A  L  V  A  G  V  S  E  R  A  M  R  H  P  S  A  M  T  S  V  F  N  A  V  D  S  I  S
817     AATGAGGTCGCTTCCATCATCCAGTCACCGGTCTCTGATGATCTTGCTATTACTGTGAAAGAGGAAAAACTTGGGGAGCTGATGGAAATGAATCAGGGTTTA
273      N  E  V  A  S  I  I  Q  S  P  V  S  D  D  L  A  I  T  V  K  E  E  K  L  G  E  L  M  E  M  N  Q  G  L
919     CTCCAGTGTATGGGGGTCAGCCATGCTACAATCGAAAACGTGATTAAAACTACTCTGAAATACAAGTTGTCGACTAAACTAACTGGAGCTGGTGGAGGGGGC
307      L  Q  C  M  G  V  S  H  A  T  I  E  N  V  I  K  T  T  L  K  Y  K  L  S  T  K  L  T  G  A  G  G  G  G
1021    TGCGTCCTCACCCTACTGCCTACGTTGTTATCAGGAACAGTGGTTGATAAACTAATTGCAGATCTAGAAGAATATGGATTCCAATGTTTGATTGCTGGAATT
341      C  V  L  T  L  L  P  T  L  L  S  G  T  V  V  D  K  L  I  A  D  L  E  E  Y  G  F  Q  C  L  I  A  G  I
1123    GGTGGAAGAGGGATGGAAATAAGCTTCAGCGGTTCTTCCTAA
375      G  G  R  G  M  E  I  S  F  S  G  S  S  *
```

Figure S5. Nucleotide sequence and deduced amino acid sequence of the *PatMVK*.

```
1       ATGGCAGTGGTGGCTTCTGCTCCTGGAAAAGTTTTGATCACTGGTGGATACCTTATTTTAGAGAGGCCTAATGCTGGGATTGTACTGAGTACAAATGCACGC
1        M  A  V  V  A  S  A  P  G  K  V  L  I  T  G  G  Y  L  I  L  E  R  P  N  A  G  I  V  L  S  T  N  A  R
103     TTCTACGCAATTGTGAAGCCACTCTATCAGGAGACTAAACCTGACAGTTGGGCATGGGCATGGACAGATGTGAAACTCACTTCTCCGCAGATGGGCAGAGAA
35       F  Y  A  I  V  K  P  L  Y  Q  E  T  K  P  D  S  W  A  W  A  W  T  D  V  K  L  T  S  P  Q  M  G  R  E
205     ACTATGTACAAATTTTCTCTCAAGCACCTCCAGCTACAATGTGTAACTTCTAGTGATGCAAGGAACCCATTTGTTGAATATGCATTGCAATATGCTGTCGCA
69       T  M  Y  K  F  S  L  K  H  L  Q  L  Q  C  V  T  S  S  D  A  R  N  P  F  V  E  Y  A  L  Q  Y  A  V  A
307     GCAGCACATGCCACATTTGATGACAGTAAGAATGACGAATTGCAGAAGCTTCTGCTGCTAGGTATTGACATTACGATCTTAGGTTGCAATGAGTTCTATTCA
103      A  A  H  A  T  F  D  D  S  K  N  D  E  L  Q  K  L  L  L  L  G  I  D  I  T  I  L  G  C  N  E  F  Y  S
409     TATAGGAATCAGATTGAAGCACGTGGCTTGCCATTGACTCCGGACTCATTGGCATCCCTTCCACCATTTTCTTCAATTTCCTTAAATGATGAAGAATCAAGT
137      Y  R  N  Q  I  E  A  R  G  L  P  L  T  P  D  S  L  A  S  L  P  P  F  S  S  I  S  L  N  D  E  E  S  S
511     GGATTAAAAAGCAAACCTGAAGTTGCTAAAACAGGATTGGGCTCATCAGCAGCTATGACTAGTGCAGTTGTTGCAGCCTTACTTAGTTACCTTGGAGTAGTC
171      G  L  K  S  K  P  E  V  A  K  T  G  L  G  S  S  A  A  M  T  S  A  V  V  A  A  L  L  S  Y  L  G  V  V
613     AATCTTCCTTCAGAGTTAAATAGTCCTTTTCAAGAGAGTAGAGTTTCCGAAGAACTAGATATTGTGCATATCATTGCTCAAACCGCTCACTGTATAGCCCAA
205      N  L  P  S  E  L  N  S  P  F  Q  E  S  R  V  S  E  E  L  D  I  V  H  I  I  A  Q  T  A  H  C  I  A  Q
715     GGCAAAGTTGGTAGTGGTTTCGATGTGAGTTCTGCTGTTTATGGTAGTCAGCGGTATATCAGATTTTCACCAGAAGTACTTTCTTCTGCTCAGGATGTTTCT
239      G  K  V  G  S  G  F  D  V  S  S  A  V  Y  G  S  Q  R  Y  I  R  F  S  P  E  V  L  S  S  A  Q  D  V  S
817     CATGGGATGCCAATGGAAGAGGTCATAGGAAAGGTGCTGAAGGACAAATGGGATCATGAGAGGACTAAGTTTTCATTGCCTCCAATGATGACATTACTTCTT
273      H  G  M  P  M  E  E  V  I  G  K  V  L  K  D  K  W  D  H  E  R  T  K  F  S  L  P  P  M  M  T  L  L  L
919     GGAGAACCAGGAGCTGGTGGATCATCAACCCCTTCAATGGTTGGTGCTGTAAAGAAGTGGCAAAAGTCTGACCCCAAAAGCTCTCTTAAAACATGGAAGAAG
307      G  E  P  G  A  G  G  S  S  T  P  S  M  V  G  A  V  K  K  W  Q  K  S  D  P  K  S  S  L  K  T  W  K  K
1021    CTGTCAGAGGCAAATTCTGCTCTCGAAAGGCACCTCAAGACCTTGAGCAAACTGGCAGAAACAAATTATGATGATTATAGAAGTTCCATCAGCAAATGCAGC
341      L  S  E  A  N  S  A  L  E  R  H  L  K  T  L  S  K  L  A  E  T  N  Y  D  D  Y  R  S  S  I  S  K  C  S
1123    ATGCTAACATACAAAAAGACAGAGGGAGCTATTGAACCAAATCATATAGAAGTGGTTAAAGCACTATTAGGGGCCAGAGATGCTATGCTTATGATCAGGTGT
375      M  L  T  Y  K  K  T  E  G  A  I  E  P  N  H  I  E  V  V  K  A  L  L  G  A  R  D  A  M  L  M  I  R  C
1225    AACATGCACAGGATGGGCGAGGCTGCTGGAGTTCCTATCGAACCCGACTCACAAACTAAACTGCTGGATACCACTATGGATATGGAAGGTGTTCTTTTAGCT
409      N  M  H  R  M  G  E  A  A  G  V  P  I  E  P  D  S  Q  T  K  L  L  D  T  T  M  D  M  E  G  V  L  L  A
1327    GGTGTTCCTGGTGCTGGTGGTTTCGATGCAGTATTTGCTGTCACATTGGGGGATGCTAGCACCAACGTGATCAAGGTCTGGAGTTCACTCAACGTTCTCGCC
443      G  V  P  G  A  G  G  F  D  A  V  F  A  V  T  L  G  D  A  S  T  N  V  I  K  V  W  S  S  L  N  V  L  A
1429    TTGCTAGTAAATGAGGATCCTCGTGGCGTCTCCCTAGAGAGCCACGACCCACGAACTACAGAAATAACTGGTGCTGTTGCTTCCATTCGTATTGAGTGA
477      L  L  V  N  E  D  P  R  G  V  S  L  E  S  H  D  P  R  T  T  E  I  T  G  A  V  A  S  I  R  I  E  *
```

Figure S6. Nucleotide sequence and deduced amino acid sequence of the *PatPMK*.

```
1     ATGGCGGCGGAGGGCGGAGAGAAATGGATTCTCACTGTCACGGCGCAGACACCCACCAACATAGCGGTGATTAAGTACTGGGGAAAGAGAGACGAAGACCTC
1      M  A  A  E  G  G  E  K  W  I  L  T  V  T  A  Q  T  P  T  N  I  A  V  I  K  Y  W  G  K  R  D  E  D  L
103   ATTCTTCCGATTAATGACAGTATTAGCGTTACTCTCGACCCCGACCACCTCTGCACCACCACCTCCGTCGCCGTTAGCCCCTCTTTCACTCATGATCGTATG
35     I  L  P  I  N  D  S  I  S  V  T  L  D  P  D  H  L  C  T  T  T  S  V  A  V  S  P  S  F  T  H  D  R  M
205   TGGCTCAACGGAAAGGAGGTATCTCTTTCTGGAGGCAGATATCAAAATTGCTTGAGGGAACTTCGCTCACGTGCTAGCGATTTTGAGGATGAGAAAAAGGGT
69     W  L  N  G  K  E  V  S  L  S  G  G  R  Y  Q  N  C  L  R  E  L  R  S  R  A  S  D  F  E  D  E  K  K  G
307   ATTAAGATAACAAAAAAGGACTGGGAGAAGCTGCATGTGCATGTTGTTTCATATAACAATTTTCCGACCGCTGCTGGTCTGGCATCATCAGCTGCTGGTTTA
103    I  K  I  T  K  K  D  W  E  K  L  H  V  H  V  V  S  Y  N  N  F  P  T  A  A  G  L  A  S  S  A  A  G  L
409   GCATGCCTTGTTTTCTCCCTGGCTAAGCTAATGAATGTGAAAGAAGATCACAGCAAACTGTCTGCTATTGCAAGGCAAGGTTCAGGAAGTGCTTGCCGCAGC
137    A  C  L  V  F  S  L  A  K  L  M  N  V  K  E  D  H  S  K  L  S  A  I  A  R  Q  G  S  G  S  A  C  R  S
511   CTGTATGGTGGATTTGTCAAATGGATCATGGGAAAAGAGCAAGATGGAAGTGATAGCATTGCTGTTCAGCTTACCGATGAGAAGCACTGGGAGGACCTTGTT
171    L  Y  G  G  F  V  K  W  I  M  G  K  E  Q  D  G  S  D  S  I  A  V  Q  L  T  D  E  K  H  W  E  D  L  V
613   ATTATCATTGCAGTGGTAAGTTCAAGACAGAAGGAAACCAGTAGTACCTCCGGAATGCGTGAGACTGTTGAAACCAGCGAACTTATAAAACATAGAGCACAG
205    I  I  I  A  V  V  S  S  R  Q  K  E  T  S  S  T  S  G  M  R  E  T  V  E  T  S  E  L  I  K  H  R  A  Q
715   GAAGTAGTTCCTAAACGTATCATAAAAATGGAAGAAGCAATCGCAAAGCGTGATTTTCCTGCTTTTGCTCATCTGACATGTGCAGACAGCAATCAGTTTCAT
239    E  V  V  P  K  R  I  I  K  M  E  E  A  I  A  K  R  D  F  P  A  F  A  H  L  T  C  A  D  S  N  Q  F  H
817   GCAGTTTGCCTTGATACTTTACCCCCCATATTCTACATGAATGACACTTCTCATAAGATAATCAGCTGTGTTGAGAAATGGAATCGTCATGAAGGATCACCT
273    A  V  C  L  D  T  L  P  P  I  F  Y  M  N  D  T  S  H  K  I  I  S  C  V  E  K  W  N  R  H  E  G  S  P
919   CAGGTGGCGTATACCTTCGATGCTGGGCCGAATGCAGTACTAATTTCACATAACAGAAAAACTGCTACCCTTCTGCTTCAGAGGCTTCTCTATTACTTTCCC
307    Q  V  A  Y  T  F  D  A  G  P  N  A  V  L  I  S  H  N  R  K  T  A  T  L  L  L  Q  R  L  L  Y  Y  F  P
1021  CCTCAATCAGATACTGATTTGGACAGTTATGTTATTGGTGACAAGACGATACTAAAAGACGCTGGCATTCATGAAATGAAGGATGTGGAAGCTTTGAGTCCA
341    P  Q  S  D  T  D  L  D  S  Y  V  I  G  D  K  T  I  L  K  D  A  G  I  H  E  M  K  D  V  E  A  L  S  P
1123  CCTCCAGAAACCAAGGATAATACTCAGAGAAACAAGGGTGATGTTAGTTACTTTATCTGCACGAGGCCTGGGAGAGGTCCCATTGTGCTTACTGATGAAAGT
375    P  P  E  T  K  D  N  T  Q  R  N  K  G  D  V  S  Y  F  I  C  T  R  P  G  R  G  P  I  V  L  T  D  E  S
1225  CGGTCACTCATCAACCCAGAAACTGGCTTTCCCAAGTGA
409    R  S  L  I  N  P  E  T  G  F  P  K  *
```

Figure S7. Nucleotide sequence and deduced amino acid sequence of the *PatMVD*.

```
1     ATGGCTTTGTGCTCATTTGCATTTACTGGAAATTTGGGCAAAGGATTAGCAGCTACTGAAGCTCAAAAGAACACCAGTTTACTCTCTCACTGGCTCTATGGA
1      M  A  L  C  S  F  A  F  T  G  N  L  G  K  G  L  A  A  T  E  A  Q  K  N  T  S  L  L  S  H  W  L  Y  G
103   TCAGATCTACACTCTCTCCATTTCTCCAAGAACAATCAGGTAAGGAAAAGCTCAACCGGAATATGCGCATCACTGTCGGAAAGAGGGGAGTATTTCTCCCAG
35     S  D  L  H  S  L  H  F  S  K  N  N  Q  V  R  K  S  S  T  G  I  C  A  S  L  S  E  R  G  E  Y  F  S  Q
205   AAACCTCGTACTCCTCTTTTAGACACTATAAACTATCCAATTCACATGAAAAACCTCACTGCTAAGGAACTGAAACAACTGGCTGATGAGCTGCGGTCTGAG
69     K  P  R  T  P  L  L  D  T  I  N  Y  P  I  H  M  K  N  L  T  A  K  E  L  K  Q  L  A  D  E  L  R  S  E
307   GTGATTTTCAATGTGTCCAAGACTGGAGGCCACCTCGGCTCAAGCCTGGGTGTTATTGAGCTAACTGTGGCACTTCATTATGTATTCAATGCCCCTCAGGAT
103    V  I  F  N  V  S  K  T  G  G  H  L  G  S  S  L  G  V  I  E  L  T  V  A  L  H  Y  V  F  N  A  P  Q  D
409   AGAATTCTTTGGGATGTTGGCCATCAGGCTTATCCACATAAGATTCTTACCGGGAGAAGAGATAAGATGTCAACTTTAAGACAAACTGGTGGTCTTTCTGGT
137    R  I  L  W  D  V  G  H  Q  A  Y  P  H  K  I  L  T  G  R  R  D  K  M  S  T  L  R  Q  T  G  G  L  S  G
511   TTTACTAAGAGATCTGAGAGTGATTATGATTGCTTCGGTGCTGGTCACAGTTCTACCACCATCTCTGCTGGATTGGGAATGGCTGTGGGGAGGGACTTAAAA
171    F  T  K  R  S  E  S  D  Y  D  C  F  G  A  G  H  S  S  T  T  I  S  A  G  L  G  M  A  V  G  R  D  L  K
613   GGAAGAAAGAATAATGTTGTCGCTGTGATAGGGGATGGTGCAATGACAGCAGGTCAAGCATACGAAGCCATGAATAATGCTGGTTACCTAGACTCTGATATG
205    G  R  K  N  N  V  V  A  V  I  G  D  G  A  M  T  A  G  Q  A  Y  E  A  M  N  N  A  G  Y  L  D  S  D  M
715   ATCGTTATCCTCAACGACAACAAACAAGTTTCTTTACCTACGGCTAATTTAGATGGTCCAACTCCTCCTGTTGGTGCCTTGAGCAGTGCCTTGAGTAGGTTA
239    I  V  I  L  N  D  N  K  Q  V  S  L  P  T  A  N  L  D  G  P  T  P  P  V  G  A  L  S  S  A  L  S  R  L
817   CAGTCAAACAGACCTCTCAGAGAACTGAGAGAAGTTGCCAAGGGAGTCACCAAGCAGATTGGAGGTCCTATGCATGAATTAGCAGCAAAAGTTGATGAATAT
273    Q  S  N  R  P  L  R  E  L  R  E  V  A  K  G  V  T  K  Q  I  G  G  P  M  H  E  L  A  A  K  V  D  E  Y
919   GCTCGTGGGTTGATCAGTGGTTCAGGATCTACGCTGTTTGAAGAGCTCGGACTTTATTATATCGGTCCTGTTGATGGTCACAACATCGATGATCTCACCGCA
307    A  R  G  L  I  S  G  S  G  S  T  L  F  E  E  L  G  L  Y  Y  I  G  P  V  D  G  H  N  I  D  D  L  T  A
1021  ATTCTTAGAGAAGTCAAGAGTACTAAGACAACAGGTCCAGTGTTGATCCATGTTGTGACTGAGAAAGGCAGGGGATATCCTTACGCAGAAAAAGCTGCAGAC
341    I  L  R  E  V  K  S  T  K  T  T  G  P  V  L  I  H  V  V  T  E  K  G  R  G  Y  P  Y  A  E  K  A  A  D
1123  AAATACCATGGAGTGACCAAGTTTGATCCAGCGACTGGAAAGCAATTTAAATCGAGTGCTCCAACTCGAGCTTACACAACCTACTTCGCGGAGGCTCTTATT
375    K  Y  H  G  V  T  K  F  D  P  A  T  G  K  Q  F  K  S  S  A  P  T  R  A  Y  T  T  Y  F  A  E  A  L  I  |
1225  GCAGAAGCTGAAGTAGACAAGGATATAGTGGCAATCCATGCTGCAATGGGAGGTGGGACGGGTTTGAACCTCTTCGAACGCCGTTTCCCAAACAGATGTTTC
409    A  E  A  E  V  D  K  D  I  V  A  I  H  A  A  M  G  G  G  T  G  L  N  L  F  E  R  R  F  P  N  R  C  F
1327  GATGTTGGGATAGCAGAACAACACGCTGTAACTTTTGCTGCAGGTTTGGCTTGTGAAGGCATCAAACCGTTCTGTGCAATCTACTCATCCTTCTTGCAAAGA
443    D  V  G  I  A  E  Q  H  A  V  T  F  A  A  G  L  A  C  E  G  I  K  P  F  C  A  I  Y  S  S  F  L  Q  R
1429  GGATATGACCAGGTAGTGCATGATGTTGATTTGCAGAAGCTACCTGTGAGGTTTGCTATGGACAGAGCTGGCTTAGTAGGAGCAGATGGCCCTACACATTGT
477    G  Y  D  Q  V  V  H  D  V  D  L  Q  K  L  P  V  R  F  A  M  D  R  A  G  L  V  G  A  D  G  P  T  H  C
1531  GGGGCTTTTGATGTTGCTTACATGGCTTGCCTTCCCAACATGGTGGTGATGGCTCCTTCGGATGAGGTTGAACTATTTCACATGGTTGCAACTGCTGCAGCC
511    G  A  F  D  V  A  Y  M  A  C  L  P  N  M  V  V  M  A  P  S  D  E  V  E  L  F  H  M  V  A  T  A  A  A
1633  ATAGATGATAGACCAAGCTGCTTCCGTTATCCGAGAGGTAACGGCGTTGGTTTAGAGTTGCCACCAGGAAACAAAGGCATTCCCCTTGAGATTGGGAAAGGC
545    I  D  D  R  P  S  C  F  R  Y  P  R  G  N  G  V  G  L  E  L  P  P  G  N  K  G  I  P  L  E  I  G  K  G
1735  CGTATATTGATTGAAGGGGAGAGGGTGGCTCTCTTAGGCTATGGAGCAGCAGTACAGAGCTGTTTGGCTGCAGCTGGATTGGTAGAATCAAAGGGTTTACGG
579    R  I  L  I  E  G  E  R  V  A  L  L  G  Y  G  A  A  V  Q  S  C  L  A  A  A  G  L  V  E  S  K  G  L  R
1837  TTGACAGTTGCTGATGCTCGTTTTTGTAAGCCTTTGGATCATGCTCTTATTCGAAGCTTGGCCAAATCACACGAGGTCTTGATCACTGTGGAAGAAGGGTCG
613    L  T  V  A  D  A  R  F  C  K  P  L  D  H  A  L  I  R  S  L  A  K  S  H  E  V  L  I  T  V  E  E  G  S
1939  ATTGGTGGTTTTGGATCTCATGTAGCTCAGTTTATGGCTTTGGATGGGCTTCTTGATGGCAAGTTAAAGTGGAGACCATTGGTTCTTCCAGATCGCTACATC
647    I  G  G  F  G  S  H  V  A  Q  F  M  A  L  D  G  L  L  D  G  K  L  K  W  R  P  L  V  L  P  D  R  Y  I
2041  GATCATGGATCCCCAGCTGATCAAGTGATGGAAGCAGGGTTGTCCCCTTCTCACATTGCAGCAACAGTTTTCAATATACTGGGGAAAGCTAGGGAGGCCCTA
681    D  H  G  S  P  A  D  Q  V  M  E  A  G  L  S  P  S  H  I  A  A  T  V  F  N  I  L  G  K  A  R  E  A  L
2143  GAGATAATGTCCTAA
715    E  I  M  S  *
```

Figure S8. Nucleotide sequence and deduced amino acid sequence of the *PatDXS*.

```
1     ATGGCTCTCAATTTGCTATCTCCTACTGAAATCAAGACCCTTTCCTTCTTAGAAACCTCCAAATCCAATTACAACCTCAATCCTCTCAAATTCCAAGGTGGA
1      M  A  L  N  L  L  S  P  T  E  I  K  T  L  S  F  L  E  T  S  K  S  N  Y  N  L  N  P  L  K  F  Q  G  G
103   TTACCTATCAAGAGGAAGGTAAATAGATGCACAGCTGCAAAGAGAGTTCATTGTTCCGTGCAGCCGCCTCTTCCGGCTTGGCCTGGTCGGGCTGTTGTTGAG
35     L  P  I  K  R  K  V  N  R  C  T  A  A  K  R  V  H  C  S  V  Q  P  P  L  P  A  W  P  G  R  A  V  V  E
205   CCCGACCGCAAGAAATGGGATGGTCCTAAACCCATTTCGGTTATTGGATCCACTGGCTCCATTGGAACACAGACATTGGACATAGTTGCTGAAAATCCAGAT
69     P  D  R  K  K  W  D  G  P  K  P  I  S  V  I  G  S  T  G  S  I  G  T  Q  T  L  D  I  V  A  E  N  P  D
307   AAGTTTAGAGTTGTTGCACTTGCAGCCGGTTCAAACGTGACCCTTCTTGCTGATCAGGTGAAGAATTTTAAACCGCAATTAGTTTCAGTTCGAGATGAGTCA
103    K  F  R  V  V  A  L  A  A  G  S  N  V  T  L  L  A  D  Q  V  K  N  F  K  P  Q  L  V  S  V  R  D  E  S
409   TTAGTTAACGAACTTGAAGAGGCTCTGTCGGACGTTAAAGACAAACCTGAGATCATTCCTGGAGAGCAGGGGATGATCGAGGTCGCTCGACATCCAGATGCT
137    L  V  N  E  L  E  E  A  L  S  D  V  K  D  K  P  E  I  I  P  G  E  Q  G  M  I  E  V  A  R  H  P  D  A
511   GCTACTGTTGTTACTGGAATTGTCGGCTGTGCTGGTTTGAAGCCAACAGTGGCTGCCATAGAAGCTGGAAAAGACATTGCTTTAGCCAATAAAGAGACACTA
171    A  T  V  V  T  G  I  V  G  C  A  G  L  K  P  T  V  A  A  I  E  A  G  K  D  I  A  L  A  N  K  E  T  L
613   ATTGCTGGAGGCCCTTTCGTCCTCCCGCTTGCACACAAGCATAACGTCAAGATTCTTCCTGCAGATTCTGAACATTCTGCTATATTTCAGTGTATCCAAGGC
205    I  A  G  G  P  F  V  L  P  L  A  H  K  H  N  V  K  I  L  P  A  D  S  E  H  S  A  I  F  Q  C  I  Q  G
715   TTGCCCGAAGGTGCTCTGAGGCGTATAATTTTAACTGCATCCGGGGGTGCTTTCAGAGATTTGCCGGTTGAAAAATTAAAAGATGTGAAAGTAGCAGACGCT
239    L  P  E  G  A  L  R  R  I  I  L  T  A  S  G  G  A  F  R  D  L  P  V  E  K  L  K  D  V  K  V  A  D  A
817   CTAAAGCATCCTAACTGGAATATGGGGAAAAAGATTACAGTAGACTCTGCAACACTCTTTAACAAGGGTTTAGAAGTTATAGAAGCTCACTATCTGTATGGG
273    L  K  H  P  N  W  N  M  G  K  K  I  T  V  D  S  A  T  L  F  N  K  G  L  E  V  I  E  A  H  Y  L  Y  G
919   GCCGACTATGACGATATAGAGATTATTATTCATCCTCAATCTATCATACACTCAATGGTTGAGACACAAGATTCATCTGTACTAGCGCAACTAGGATGGCCT
307    A  D  Y  D  D  I  E  I  I  I  H  P  Q  S  I  I  H  S  M  V  E  T  Q  D  S  S  V  L  A  Q  L  G  W  P
1021  GATATGCGTTTACCTATTCTATACACGTTATCATGGCCGGAGAGAATCTACTGCTCCGAGATTACTTGGCCCCGTCTTGATCTTTGCAAGGTCTCTCTTACA
341    D  M  R  L  P  I  L  Y  T  L  S  W  P  E  R  I  Y  C  S  E  I  T  W  P  R  L  D  L  C  K  V  S  L  T
1123  TTCAAGGCGCCAGACAATGTCAAATACCCTTCGATGGATCTAGCTTATGCTGCTGGGCGAGCTGGGGGGACCATGACCGGTGTTCTCAGTGCAGCTAATGAG
375    F  K  A  P  D  N  V  K  Y  P  S  M  D  L  A  Y  A  A  G  R  A  G  G  T  M  T  G  V  L  S  A  A  N  E
1225  AAAGCCGTCGAAATGTTCATCAACGAGCAAATTGGTTACCTCGACATATTCAAGGTCGTAGAGCTGACATGCGATAAGCATCGAGAAGATCTTATCGTCTCG
409    K  A  V  E  M  F  I  N  E  Q  I  G  Y  L  D  I  F  K  V  V  E  L  T  C  D  K  H  R  E  D  L  I  V  S
1327  CCTTCTCTGGAAGAAATCATCCACTACGACTTGTGGGCGCGGGATTACGCGGCCAGCTTACAGCAATCGACCGGTCCTCGTCCCGCGCTTGTGTGA
443    P  S  L  E  E  I  I  H  Y  D  L  W  A  R  D  Y  A  A  S  L  Q  Q  S  T  G  P  R  P  A  L  V  *
```

Figure S9. Nucleotide sequence and deduced amino acid sequence of the *PatDXR*.

```
1      ATGTCAATGCTTCAAGTGTGCAATCTACCCTCTTCAACCTCTCTCTCTTCGTCCCCTTTTTTCTTTAGGCCATTTCTGGGAGCTAATTACCTTGTTCCCAGA
1       M  S  M  L  Q  V  C  N  L  P  S  S  T  S  L  S  S  S  P  F  F  F  R  P  F  L  G  A  N  Y  L  V  P  R
103    AAACTGCACCCTAACCATATCAAGAACCTCGCTTTCACTTCAAGAATCAAGAACCCAACTTTTTTAAGAATCAGTTGTTCAACTAAAGGTGAAGAGGGGTCT
35      K  L  H  P  N  H  I  K  N  L  A  F  T  S  R  I  K  N  P  T  F  L  R  I  S  C  S  T  K  G  E  E  G  S
205    GGTGTATTGGTCAAAGAGAAAAGTGTCGCTGTTGTATTGCTTGCAGGAGGAAAGGGCAAGAGAATGGGTGCGAACATGCCAAAGCAGTATCTTCCACTTCTT
69      G  V  L  V  K  E  K  S  V  A  V  V  L  L  A  G  G  K  G  K  R  M  G  A  N  M  P  K  Q  Y  L  P  L  L
307    GGCCAACCGATTGCTTTATACAGTTTCTATACTTTCTCTATGATGCCTGAAGTGAAGGAAATTGTTGTCGTATGTGATCCTTCTTATCAAGACATTTTTGAA
103     G  Q  P  I  A  L  Y  S  F  Y  T  F  S  M  M  P  E  V  K  E  I  V  V  V  C  D  P  S  Y  Q  D  I  F  E
409    GATGCCAAAGAGAGTGTCTCCATTGACCTGAAATTTGCATTACCAGGGAAGGAGAGACAAGATTCTGTATACAGTGGATTAGAGGCAATTGATTCAAATTCC
137     D  A  K  E  S  V  S  I  D  L  K  F  A  L  P  G  K  E  R  Q  D  S  V  Y  S  G  L  E  A  I  D  S  N  S
511    GAGCTAGTTTGCATACATGACTCTGCAAGACCTCTCGTGCTAGCTGCAGATGTAGAGAAGGTTCTGAAGGATGGTAGGCGTGTCGGTGCAGCTGTGCTAGGC
171     E  L  V  C  I  H  D  S  A  R  P  L  V  L  A  A  D  V  E  K  V  L  K  D  G  R  R  V  G  A  A  V  L  G
613    GTTCCTGCTAAAGCTACAATCAAGGAGGCAAATAGTGAATCTTTTGTCGTAAAAACTCTGGACAGGAAAACACTCTGGGAAATGCAAACTCCACAAGTAATC
205     V  P  A  K  A  T  I  K  E  A  N  S  E  S  F  V  V  K  T  L  D  R  K  T  L  W  E  M  Q  T  P  Q  V  I
715    AAGCCTGATTTGCTTAAGAAAGGCTTCGAACTTGTTAATAGGGAAGGACTTGAAGTTACAGATGATGTATCAATTGTGGAGCACCTTAAACATCCTGTGTTC
239     K  P  D  L  L  K  K  G  F  E  L  V  N  R  E  G  L  E  V  T  D  D  V  S  I  V  E  H  L  K  H  P  V  F
817    ATTACACATGGATCTTACACCAACATCAAGGTTACCACCCCAGATGATCTCTTACTTGCTGAGAGAATATTGAATCCCGCTAGCTGA
273     I  T  H  G  S  Y  T  N  I  K  V  T  T  P  D  D  L  L  L  A  E  R  I  L  N  P  A  S  *
```

Figure S10. Nucleotide sequence and deduced amino acid sequence of the *PatMCT*.

```
1       ATGGCTTCCTCCCATTCCCTCTGCAGCCACCAGAGATACACCTCCTGTAATCCAAGAACCCAGTTTAATTCCTTCAAGATTCCCAATTTTTCTTCACCTTCT
1        M  A  S  S  H  S  L  C  S  H  Q  R  Y  T  S  C  N  P  R  T  Q  F  N  S  F  K  I  P  N  F  S  S  P  S
103     TCGTTTAAGCCACATGGGTCTTCCTCTTACCCCAGAAAATTTCAGTCTTTGATCAGAGCGGCTGCTTCTGATTCTAAATCCGGTACAAAGCAAGTAGAGGTG
35       S  F  K  P  H  G  S  S  S  Y  P  R  K  F  Q  S  L  I  R  A  A  A  S  D  S  K  S  G  T  K  Q  V  E  V
205     GTGTATGATTTTGAGGGTAAGCTTAATAAGTTAGCTGATGAAGTGGATATGGATGCTGGGATTTCAAGACTCACTCTATATTCCCCTTGCAAGATCAACGTT
69       V  Y  D  F  E  G  K  L  N  K  L  A  D  E  V  D  M  D  A  G  I  S  R  L  T  L  Y  S  P  C  K  I  N  V
307     TTCCTGAGAATAACTGGAAAACGAACAGATGGTTATCATGATTTGGCATCCCTCTTTCATGTAATCAGTTTAGGAGATAAAATAAAGTTTTCACTGTCGCCA
103      F  L  R  I  T  G  K  R  T  D  G  Y  H  D  L  A  S  L  F  H  V  I  S  L  G  D  K  I  K  F  S  L  S  P
409     TCAAAATCAAAGGATGGTTTGTCAACCAATGTCCCGGGAGTTCCTCTTGATGACAAAAATTTGATAATAAAGGCCCTTAACCTCTTCAGAAAAAAAACAGGG
137      S  K  S  K  D  G  L  S  T  N  V  P  G  V  P  L  D  D  K  N  L  I  I  K  A  L  N  L  F  R  K  K  T  G
511     ATAAACAACTACTTTTGGGTTCATCTTGATAAGAAAGTACCTACAGGGGCAGGCCTTGGTGGGGGCAGCGGCAATGCCGCTACTGCTTTGTGGGCAGCAAAT
171      I  N  N  Y  F  W  V  H  L  D  K  K  V  P  T  G  A  G  L  G  G  G  S  G  N  A  A  T  A  L  W  A  A  N
613     CAGTTTAGTGGTTGTGTTGCTACTGAAAAGGATCTCCAAGATTGGTCTGGTGAGATTGGATCTGATATCCCATTCTTTTTCTCTCACGGAGCTGCATATTGT
205      Q  F  S  G  C  V  A  T  E  K  D  L  Q  D  W  S  G  E  I  G  S  D  I  P  F  F  F  S  H  G  A  A  Y  C
715     ACAGGGAGAGGTGAGGTAGTTGAAGATATATCTTCCCCTATACCTGTTGATCTTCCTATGGTTCTCATTAAACCGCAAGAGGCTTGTCCAACTGCTGAAGTT
239      T  G  R  G  E  V  V  E  D  I  S  S  P  I  P  V  D  L  P  M  V  L  I  K  P  Q  E  A  C  P  T  A  E  V
817     TACAAGCGTCTGCGGCTCGAGCAAACAAGCAGTGTGGATCCTTTGTTGCTGCTAGAGAAGATTTCAAAGAATGGAATTTCTCAGGAGGTCTGCGTCAATGAT
273      Y  K  R  L  R  L  E  Q  T  S  S  V  D  P  L  L  L  L  E  K  I  S  K  N  G  I  S  Q  E  V  C  V  N  D
919     CTTGAACCTCCTGCTTTTGAAGTTCTTCCATCACTAAAAAGATTAAAGCAACGGATAGCTGCTGCAGGTAGAGGGCAATATGATGCTGTTTTCATGTCTGGA
307      L  E  P  P  A  F  E  V  L  P  S  L  K  R  L  K  Q  R  I  A  A  A  G  R  G  Q  Y  D  A  V  F  M  S  G
1021    AGTGGGAGTACTATTGTTGGGGTGGGTTCTCCCGACCCCCCACTATTTGTCTACGAAGATGACGAGTACAAAGATGTCTTCTTATCAGAAGCAAGATTCATC
341      S  G  S  T  I  V  G  V  G  S  P  D  P  P  L  F  V  Y  E  D  D  E  Y  K  D  V  F  L  S  E  A  R  F  I
1123    ACCCGGTCAGCTAATCAGTGGTATACAGAGCCTCTCTTGACCAATGCTGATACCGAACAAACGAGGGGCTTTTCGCGCTCAGTCGAATAA
375      T  R  S  A  N  Q  W  Y  T  E  P  L  L  T  N  A  D  T  E  Q  T  R  G  F  S  R  S  V  E  *
```

Figure S11. Nucleotide sequence and deduced amino acid sequence of the *PatCMK*.

```
1      GTGGTGGCTGCTGCCTCGAGCACTGTTGAAGCCGAGCCGCAGTCGGAAGCTGCTGCCCCGGCGAAGGTTTTGCCTTTTCGCGTTGGCCACGGATTCGACCTT
1       V  V  A  A  A  S  S  T  V  E  A  E  P  Q  S  E  A  A  A  P  A  K  V  L  P  F  R  V  G  H  G  F  D  L
103    CACCGGCTCGAACCGGGTTATCCTCTAATCATCGGCGGTATCAGTATTCCACATGATCGAGGCTGCGAAGCCCATTCCGACGGTGATGTGTTGCTGCACTGT
35      H  R  L  E  P  G  Y  P  L  I  I  G  G  I  S  I  P  H  D  R  G  C  E  A  H  S  D  G  D  V  L  L  H  C
205    GTGGTTGATGCAATACTGGGGGCATTGGGGCTCCCGGATATCGGCCAAATATTTCCGGATACGGATCCTAAGTGGAAAGGTGCAGCATCTTCTGTTTTCATA
69      V  V  D  A  I  L  G  A  L  G  L  P  D  I  G  Q  I  F  P  D  T  D  P  K  W  K  G  A  A  S  S  V  F  I
307    AAGGAGGCAGTTCGGCTAATGCATGAGGCGGGCTACGAGCTTGGAAATCTGGACTCGACGTTGATTCTCCAAAGACCAAAGTTGAGCCCTCACAAGGAGGCT
103     K  E  A  V  R  L  M  H  E  A  G  Y  E  L  G  N  L  D  S  T  L  I  L  Q  R  P  K  L  S  P  H  K  E  A
409    ATAAGGGCTAATTTATGCGAGCTACTTGGAGCGGATCCCTCGGTGGTGAATCTAAAGGCTAAAACTCATGAGAAGGTCGATAGTCTCGGGGAGAACCGGAGT
137     I  R  A  N  L  C  E  L  L  G  A  D  P  S  V  V  N  L  K  A  K  T  H  E  K  V  D  S  L  G  E  N  R  S
511    ATAGCAGCACATACAGTTGTTCTTCTTATGAAGAAGTAA
171     I  A  A  H  T  V  V  L  L  M  K  K  *
```

Figure S12. Nucleotide sequence and deduced amino acid sequence of the *PatMDS*.

```
1       ATGGCAACGGGTGCTGTTCCGGCTTCATTTACTGGTGTGAAAACCAGGGATCATCGTGGCGTAGGGTTCGCTAAGACTTCGGATTTTGTTAGAATTTCTGAT
1        M  A  T  G  A  V  P  A  S  F  T  G  V  K  T  R  D  H  R  G  V  G  F  A  K  T  S  D  F  V  R  I  S  D
103     GTGCAAAGGGTTAAATTTGGTAGAACCAAGATTGCAGTGATCAGAAATTCTACCAACCCTGGTTCAGAAACTGTTGAACTTGAGCAAGCATCAGAAGGAAGC
35       V  Q  R  V  K  F  G  R  T  K  I  A  V  I  R  N  S  T  N  P  G  S  E  T  V  E  L  E  Q  A  S  E  G  S
205     CCACTGCTAGTTCCTAGACAGAAGTACTGTGAATCCATACACAAAACCATCCGGAGAAAAACCCGCACAGTGATGGTTGGAAATGTTGCACTTGGTAGTGAG
69       P  L  L  V  P  R  Q  K  Y  C  E  S  I  H  K  T  I  R  R  K  T  R  T  V  M  V  G  N  V  A  L  G  S  E
307     CATCCCATCAGGATTCAAACAATGACCACTACTGATACAAAGGATGTTGCTGCAACTGTTGATCAGGTGATGAGAATAGCAGATCAGGGAGCAGATATTGTT
103      H  P  I  R  I  Q  T  M  T  T  T  D  T  K  D  V  A  A  T  V  D  Q  V  M  R  I  A  D  Q  G  A  D  I  V
409     AGAATTACAGTACAAGGAAGGAAAGAAGCTGATGCTTGTTATGACATTAAAGATACCCTTGTTAAGAAGAACTATAACATCCCTCTGGTGGCAGACATTCAT
137      R  I  T  V  Q  G  R  K  E  A  D  A  C  Y  D  I  K  D  T  L  V  K  K  N  Y  N  I  P  L  V  A  D  I  H
511     TTTGCTCCACCTGTGGCTATGCGAGTTGCTGAATGTTTTGATAAAATTCGTGTTAACCCAGGAAACTTTGCTGATAGAAGAGCGCAGTTTGAGAAGCTTGAG
171      F  A  P  P  V  A  M  R  V  A  E  C  F  D  K  I  R  V  N  P  G  N  F  A  D  R  R  A  Q  F  E  K  L  E
613     TACACAGAAGACGATTACCAAAAAGAACTTGAGCATATTGAGAAGGTGTTCACTCCATTGGTTGAAAAATGTAAGAAGTATGGCCGAGCAATGCGTATTGGG
205      Y  T  E  D  D  Y  Q  K  E  L  E  H  I  E  K  V  F  T  P  L  V  E  K  C  K  K  Y  G  R  A  M  R  I  G
715     ACAAACCATGGTAGCCTTTCAGATCGTATAATGAGTTACTATGGGGACTCACCCAGGGGAATGGTTGAATCTGCATTCGAGTATGCTAGGATTTGTCGGAAT
239      T  N  H  G  S  L  S  D  R  I  M  S  Y  Y  G  D  S  P  R  G  M  V  E  S  A  F  E  Y  A  R  I  C  R  N
817     TTGGACTTCCACAATTTTGTGTTCTCAATGAAAGTTAGCAACCCTGTTATTATGGTCCAGGCATACCGCCTTCTTGTAGCTGAAATGAATGTACTAGGATGG
273      L  D  F  H  N  F  V  F  S  M  K  V  S  N  P  V  I  M  V  Q  A  Y  R  L  L  V  A  E  M  N  V  L  G  W
919     GATTACCCATTACATCTGGGAGTAACTGAAGCTGGTGAGGGTGAAGATGGACGAATGAAATCTGCAATAGGAATTGGGACACTTCTTATGGATGGTCTTGGT
307      D  Y  P  L  H  L  G  V  T  E  A  G  E  G  E  D  G  R  M  K  S  A  I  G  I  G  T  L  L  M  D  G  L  G
1021    GATACCATAAGGGTTTCTCTAACCGAACCTCCAGAAGAGGAGATAGATCCCTGTAGAAGATTGGCTAATCTTGGCATGAGGGCAGCTGAACTTCAGAAGGGA
341      D  T  I  R  V  S  L  T  E  P  P  E  E  E  I  D  P  C  R  R  L  A  N  L  G  M  R  A  A  E  L  Q  K  G
1123    GTGACACAATTTGAAGAAAAGCACCGACATTATTTTGACTTCCAACGCAGAAGTGGTCAACTTCCAGTTCAAAAGGAGGGTGAAGAGGTTGATTTTAGAGGA
375      V  T  Q  F  E  E  K  H  R  H  Y  F  D  F  Q  R  R  S  G  Q  L  P  V  Q  K  E  G  E  E  V  D  F  R  G
1225    GTACTTCACCGTGATGGCTCAGTGCTGATGTCTGTTTCTCTTGATCAGTTGAAGGCACCTGAACAGCTATACAAGTCTCTTGCAACAAAACTTATCGTCGGA
409      V  L  H  R  D  G  S  V  L  M  S  V  S  L  D  Q  L  K  A  P  E  Q  L  Y  K  S  L  A  T  K  L  I  V  G
1327    ATGCCATTTAAGGATTTGGCGACTGTGGATTCAATCTTGTTGAGAGAGCTTCCACCACAAGATGATAAAGATGCTAGATTGGCTCTCAAACGTTTGATAGAC
443      M  P  F  K  D  L  A  T  V  D  S  I  L  L  R  E  L  P  P  Q  D  D  K  D  A  R  L  A  L  K  R  L  I  D
1429    ATTAGTATGGGAGTAATAACTCCATTATCAGAACAGTTGACAAAGCGATTACCCAATGCAATGGCCCTGGTAACTCTAAAGGAATTGGCATCTGGTGCTCAC
477      I  S  M  G  V  I  T  P  L  S  E  Q  L  T  K  R  L  P  N  A  M  A  L  V  T  L  K  E  L  A  S  G  A  H
1531    AAGCTACTTCCAGAAGGGACACGTTTGGTCATCTCTTTGCGTGGTGACGAACCTGAAGAAGAGTTAGACGTTCTGAAGAGTGCTGATGCTACCATGATCCTT
511      K  L  L  P  E  G  T  R  L  V  I  S  L  R  G  D  E  P  E  E  E  L  D  V  L  K  S  A  D  A  T  M  I  L
1633    CATCACATACCACATGGAGAAGAAAAAATCAGCAGACTTCATGCTGCTAGAAGGCTTTTTGAGTATCTTACCAAAAACTCCTTGAACTTCGCAGTTATTCAT
545      H  H  I  P  H  G  E  E  K  I  S  R  L  H  A  A  R  R  L  F  E  Y  L  T  K  N  S  L  N  F  A  V  I  H
1735    CATATAGATTTCCCCAAACAAGTCCACAGAGATGATTTAGTTATTGGTGCCGGGACCAATGCAGGAGCTCTCCTAGTAGATGGACTCGGAGATGGTGTCCTA
579      H  I  D  F  P  K  Q  V  H  R  D  D  L  V  I  G  A  G  T  N  A  G  A  L  L  V  D  G  L  G  D  G  V  L
1837    CTAGATGCACCTGATCAAGACTTTGAATTCCTCCGAAACACTTCTTTCAATTTGCTACAAGGATGCAGAATGAGAAACACAAAGACGGAATATGTGTCATGC
613      L  D  A  P  D  Q  D  F  E  F  L  R  N  T  S  F  N  L  L  Q  G  C  R  M  R  N  T  K  T  E  Y  V  S  C
1939    CCATCCTGCGGAAGGACTTTGTTTGACCTTCAAGAGATTAGTGCAGAAATAAGAGAAAAAACATCTCATTTGCCTGGTGTTTCGATTGCAATCATGGGTTGC
647      P  S  C  G  R  T  L  F  D  L  Q  E  I  S  A  E  I  R  E  K  T  S  H  L  P  G  V  S  I  A  I  M  G  C
2041    ATTGTGAATGGGCCTGGTGAAATGGCTGATGCTGATTTTGGCTATGTTGGTGGCGCACCTGGAAAGATCGACCTTTATGTTGGAAAGACCGTGGTAAAAAGA
681      I  V  N  G  P  G  E  M  A  D  A  D  F  G  Y  V  G  G  A  P  G  K  I  D  L  Y  V  G  K  T  V  V  K  R
2143    GCTATAGAGATGGAACATGCGACGGAGGCGTTGATCGAGCTGATCAAAGAGCACGGTCGCTGGGTTGACCCACCAACAGAGGAGTAA
715      A  I  E  M  E  H  A  T  E  A  L  I  E  L  I  K  E  H  G  R  W  V  D  P  P  T  E  E  *
```

Figure S13. Nucleotide sequence and deduced amino acid sequence of the *PatHDS*.

```
1       ATGGCCATCTCTCTGCAATTCTGTCGTATCTCCGCGATCACCGAACTTCCCTTTCCGGAGACCAAGTTATTCCGGCCACGGAATCCTTCCACGCTCCGATGC
1        M  A  I  S  L  Q  F  C  R  I  S  A  I  T  E  L  P  F  P  E  T  K  L  F  R  P  R  N  P  S  T  L  R  C
103     TACTCCGTCGCCGACGCCGCTTCATCTTCCTCCGCCGAGTTTGACGCCAAGGTTTTCGTCACAACTTGACCAGGAGCAAGAATTACAACCGCAAGGGTTTT
35       Y  S  V  A  D  A  A  S  S  S  S  A  E  F  D  A  K  V  F  R  H  N  L  T  R  S  K  N  Y  N  R  K  G  F
205     GGGCATAAAGAGGAAACTCTTGAGCAAATGAGCCAAGAGTACACAAGTGACATCATAAAGAAATTGAAGGAGAATGGAAATGAATACACATGGGGAAATGTT
69       G  H  K  E  E  T  L  E  Q  M  S  Q  E  Y  T  S  D  I  I  K  K  L  K  E  N  G  N  E  Y  T  W  G  N  V
307     ACTGTGCAGCTTGCTGAAGCTTATGGATTTGCTGGGGGGTTGAGCGGGCTGTCCAGATTGCTTATGAAGCTAGAAAGCAGTTTCCAACAGAGAATATTTGG
103      T  V  Q  L  A  E  A  Y  G  F  C  W  G  V  E  R  A  V  Q  I  A  Y  E  A  R  K  Q  F  P  T  E  N  I  W
409     CTTACCAATGAAATTATTCACAACCCCACTGTCAATGAGAGGCTTCAAGATATGGAAGTCAATGAAATTCCTGTTGACAACGGGGGGAAACAATTTGGTGTT
137      L  T  N  E  I  I  H  N  P  T  V  N  E  R  L  Q  D  M  E  V  N  E  I  P  V  D  N  G  G  K  Q  F  G  V
511     GTTAACAAGGGTGATGTTGTGGTCCTGCCTGCTTTTGGAGCTTCCGTAGATGAGATGTTTCTTCTCAGTGAAAAGAATGTTCAAATAGTTGATACAACTTGC
171      V  N  K  G  D  V  V  V  L  P  A  F  G  A  S  V  D  E  M  F  L  L  S  E  K  N  V  Q  I  V  D  T  T  C
613     CTATGGGTATCTAAGGTTTGGAATACTGTTGAAAAGCACAAGAAGGGAGAATACACTTCAATAATCCATGGCAAATACTCTCATGAAGAGACTGTGGCTACT
205      L  W  V  S  K  V  W  N  T  V  E  K  H  K  K  G  E  Y  T  S  I  I  H  G  K  Y  S  H  E  E  T  V  A  T
715     GCTTCTTTTGCTGGGAAGTATATCATTGTAAAGAACATGAAAGAGGCAACCTATGTTTGTGATTACATACTGGGTGGCGGACTTGATGGATCCAGCTCAACC
239      A  S  F  A  G  K  Y  I  I  V  K  N  M  K  E  A  T  Y  V  C  D  Y  I  L  G  G  G  L  D  G  S  S  S  T
817     AAAGAGGCATTTCTGGAGAAATTTAAATTTGCGGTATCCAAAGGGTTTGATCCAGACAAAGATCTTGATAAAGTTGGTATCGCAAATCAAACTACTATGTTA
273      K  E  A  F  L  E  K  F  K  F  A  V  S  K  G  F  D  P  D  K  D  L  D  K  V  G  I  A  N  Q  T  T  M  L
919     AAGGGAGAAACAGAGGATATTGGGAAATTAGTTGAGAGGACCATGATGCGCAAGAATGGGGTCGAAAATATTAACAACCACTTCATAAGTTTCAACACTATT
307      K  G  E  T  E  D  I  G  K  L  V  E  R  T  M  M  R  K  N  G  V  E  N  I  N  N  H  F  I  S  F  N  T  I
1021    TGTGATGCTACTCAAGAACGCCAAGATGCAATGTATAAACTGGTTGACCAGGACATGGATCTCATCCTAGTTGTTGGCGGATGGAACTCAAGCAACACTTCA
341      C  D  A  T  Q  E  R  Q  D  A  M  Y  K  L  V  D  Q  D  M  D  L  I  L  V  V  G  G  W  N  S  S  N  T  S
1123    CATCTACAAGAAATTGCAGAGGAGCGCGGGATTCCATCATATTGGATTGACAGTGAAAGAAGAATAGGCCCTGGCAACAAAATTAGCCACAAGTTGATGCAT
375      H  L  Q  E  I  A  E  E  R  G  I  P  S  Y  W  I  D  S  E  R  R  I  G  P  G  N  K  I  S  H  K  L  M  H
1225    GGTGAGTTGGTGGAGAAAGAGGACTGGCTACCAAAGGGTCCTATCAAAATTGGCGTCACATCTGGTGCATCCACTCCCGACAAGGTTGTCGAAAACGCCCTT
409      G  E  L  V  E  K  E  D  W  L  P  K  G  P  I  K  I  G  V  T  S  G  A  S  T  P  D  K  V  V  E  N  A  L
1327    GTTAAGGTTTTCGATATCAAGCATGAGGAAGTATTGCAACTGGCATGAAAGGGC
443      V  K  V  F  D  I  K  H  E  E  V  L  Q  L  A  *
```

Figure S14. Nucleotide sequence and deduced amino acid sequence of the *PatHDR*.

```
1    ATGGCGATGGCGTCCAATACTATCATCCGCCTTCAAAATTCATTTGCTCTATCACCATCTGCTTCTCCTCGTTTTTCTCTCCTTCCTTCAAGGCTCAGGCAG
1     M  A  M  A  S  N  T  I  I  R  L  Q  N  S  F  A  L  S  P  S  A  S  P  R  F  S  L  L  P  S  R  L  R  Q
103  TTCCCCCTCCGCGTCTGCTCCTCTATTTCCGCCGTCACATCACTCCCCTCAGCCATGGGTGTCGACCTTTCTGCCGATTCCGCCATGGATGCGGTTCAGAGA
35    F  P  L  R  V  C  S  S  I  S  A  V  T  S  L  P  S  A  M  G  V  D  L  S  A  D  S  A  M  D  A  V  Q  R
205  CGTCTCATGTTTGATGACGAATGCATACTGGTGGATGAGAATGATCATGTGGTGGGACATGACACCAAGTACAATTGCCACCTGATGGAAAAGATCGAATCC
69    R  L  M  F  D  D  E  C  I  L  V  D  E  N  D  H  V  V  G  H  D  T  K  Y  N  C  H  L  M  E  K  I  E  S
307  GAGAACTTACTGCACAGAGCTTTCAGCGTGTTTCTATTCAACTCCAAACACGAGCTACTGCTACAGCAACGATCTTCGACGAAGGTGACCTTTCCGTTGGTG
103   E  N  L  L  H  R  A  F  S  V  F  L  F  N  S  K  H  E  L  L  L  Q  Q  R  S  S  T  K  V  T  F  P  L  V
409  TGGACCAACACTTGCTGTAGCCATCCCCTGTTCCGGGGCTCGGAGCTAATTGAGGAGAATGCTCTTGGGGTGAGGAATGCTGCTCAAAGGAAGCTGTTGGAT
137   W  T  N  T  C  C  S  H  P  L  F  R  G  S  E  L  I  E  E  N  A  L  G  V  R  N  A  A  Q  R  K  L  L  D
511  GAGCTCGGCATCCCTGCTGATGACGTCCCAGTGGATAAGTTCACTCCCTTGGGCCGTATTCTGTACAAAGCACCATCCGACGGAAAATGGGGAGAGCATGAG
171   E  L  G  I  P  A  D  D  V  P  V  D  K  F  T  P  L  G  R  I  L  Y  K  A  P  S  D  G  K  W  G  E  H  E
613  TTGGACTATCTTCTGTTCATTGTGCGGGATGTTAGCGTGCAGCCAAACCCTGATGAGGTGGCAGATGTCAAATACGTGAATCGTGACGAGTTGAAAGAGATA
205   L  D  Y  L  L  F  I  V  R  D  V  S  V  Q  P  N  P  D  E  V  A  D  V  K  Y  V  N  R  D  E  L  K  E  I
715  GTGAGAAAAGCAGATGCGGGTGAGGAGGGTTTGAAGCTCTCCCCTTGGTTTAGATTAGTGGTTGACAACTTCTTGTTCAAATGGTGGGATCATGCTGAGAAT
239   V  R  K  A  D  A  G  E  E  G  L  K  L  S  P  W  F  R  L  V  V  D  N  F  L  F  K  W  W  D  H  A  E  N
817  GGGACTTTGAAACAAGCGGCTGATATGAACACTATTCACAAGTTGATTTGA
273   G  T  L  K  Q  A  A  D  M  N  T  I  H  K  L  I  *
```

Figure S15. Nucleotide sequence and deduced amino acid sequence of the *PatIPPI*.

```
1       ATGGCCAATCCGAACGGAGCCACGGCGGATCTGCGGGAGACGTTTCTGGGGGTGTATTCGGTGCTCAAATCTGAGCTCTTGAACGACCCTGCTTTCGAGTGG
1        M  A  N  P  N  G  A  T  A  D  L  R  E  T  F  L  G  V  Y  S  V  L  K  S  E  L  L  N  D  P  A  F  E  W
103     AACGATACTTCTCGCCAATGGGTTGATCGGATGTTGGACTACAATGTTCCCGGAGGGAAGTTAAACAGAGGCCTATCTGTTATTGACAGCTATAAAATACTT
35       N  D  T  S  R  Q  W  V  D  R  M  L  D  Y  N  V  P  G  G  K  L  N  R  G  L  S  V  I  D  S  Y  K  I  L
205     AAAGAAGGAAGAGACCTCACTGAAAATGAAATATTTCTGGCTAGTGCTCTAGGCTGGTGTATTGAATGGCTTCAGGCATATTTCCTCGTCCAGGATGATATA
69       K  E  G  R  D  L  T  E  N  E  I  F  L  A  S  A  L  G  W  C  I  E  W  L  Q  A  Y  F  L  V  Q  D  D  I
307     ATGGATAACTCTCAAACACGACGTGGTCAACCATGCTGGTATAAAGTCCCTAAGGTTGGTATGATTGCTGTAAATGATGGAATCCTACTCCGGAATCATATC
103      M  D  N  S  Q  T  R  R  G  Q  P  C  W  Y  K  V  P  K  V  G  M  I  A  V  N  D  G  I  L  L  R  N  H  I
409     CCCAGAATTCTGAAGAACCATTTCAGAGAAAAGTCTTACTATGTGGATCTAGTGGATTTGTTCAATGAGGTGGAGTTCCAAACTGCTTCCGGACAGATGATA
137      P  R  I  L  K  N  H  F  R  E  K  S  Y  Y  V  D  L  V  D  L  F  N  E  V  E  F  Q  T  A  S  G  Q  M  I
511     GATTTAATTACCACTATTGAAGGGGAAAAAGATTTGTCAAAATACTCATTGCCACTTCACCGTCGTATTGTACAGTACAAGACTTCCTACTACTCGTTTTAC
171      D  L  I  T  T  I  E  G  E  K  D  L  S  K  Y  S  L  P  L  H  R  R  I  V  Q  Y  K  T  S  Y  Y  S  F  Y
613     CTTCCAGTTGCATGTGCACTGCTCATGGCGGGTGAGGACCTGGAGAAACATACAAATGTGAGAGATGTGCTTGTCAATATGGGCATTTACTTTCAAGTACAG
205      L  P  V  A  C  A  L  L  M  A  G  E  D  L  E  K  H  T  N  V  R  D  V  L  V  N  M  G  I  Y  F  Q  V  Q
715     GACGACTACTTGGATTGCTTCGGTGAGCCTGAAAAGATTGGGAAGATTGGAACAGACATAGAAGATTTCAAGTGTTCTTGGCTGGTTGTAAAAGCCCTGGAG
239      D  D  Y  L  D  C  F  G  E  P  E  K  I  G  K  I  G  T  D  I  E  D  F  K  C  S  W  L  V  V  K  A  L  E
817     CTCTGCAATGATGAACAAAAGAAAATTCTTTTCGAGCACTACGGAAAAGAAAATCCAGCTGATGTTGCAAAAATAAAAGCCCTCTACAACGAGATCAATCTC
273      L  C  N  D  E  Q  K  K  I  L  F  E  H  Y  G  K  E  N  P  A  D  V  A  K  I  K  A  L  Y  N  E  I  N  L
919     CAAGGTGCGTTTGCTGAGTATGAAAGCAAGAGCTACGAGAGCCTCACGAGCTCTATTGAAGCTCATCCTAGCAAATCGGTGCAGGCAGTGCTGAAGTCCTTC
307      Q  G  A  F  A  E  Y  E  S  K  S  Y  E  S  L  T  S  S  I  E  A  H  P  S  K  S  V  Q  A  V  L  K  S  F
1021    TTGGGCAAGATTTACAAGAGACAGAAATAA
341      L  G  K  I  Y  K  R  Q  K  *
```

Figure S16. Nucleotide sequence and deduced amino acid sequence of the *PatFPPS*.

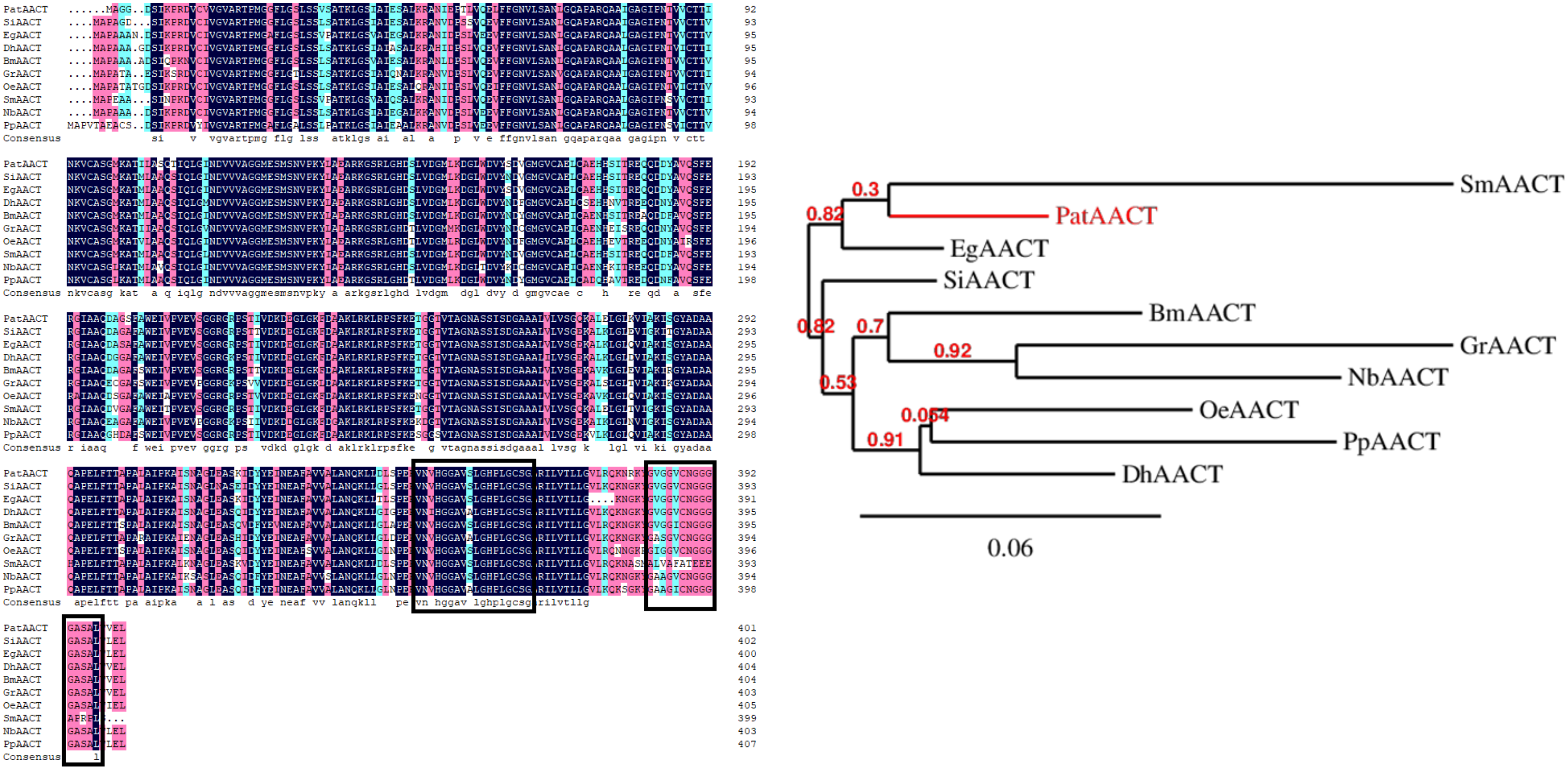

Figure S17.Sequence alignment and phylogenetic relationships of AACT proteins from *P. cablin* and various other plants. Proteins included are SiAACT (Sesamum indicum, XM 011102210.2), EgAACT (Erythranthe guttata, XM 012975391.1), DhAACT (Dorcoceras hygrometricum, KZV31357.1), BmAACT (Bacopa monnieri, FJ947159.3), GrAACT (Gentiana rigescens, AJT35503.1), OeAACT (Olea europaea var. sylvestris, XM 023030174.1), NbAACT (Nicotiana benthamiana, NM 001325842.1), PpAACT (Prunus persica, XP 007215468.1), and *P. cablin* PatAACT. The peroxisomal targeting signal 1 (PTS1) related motif (SAL) presented in the carboxy terminus of AACT is boxed.

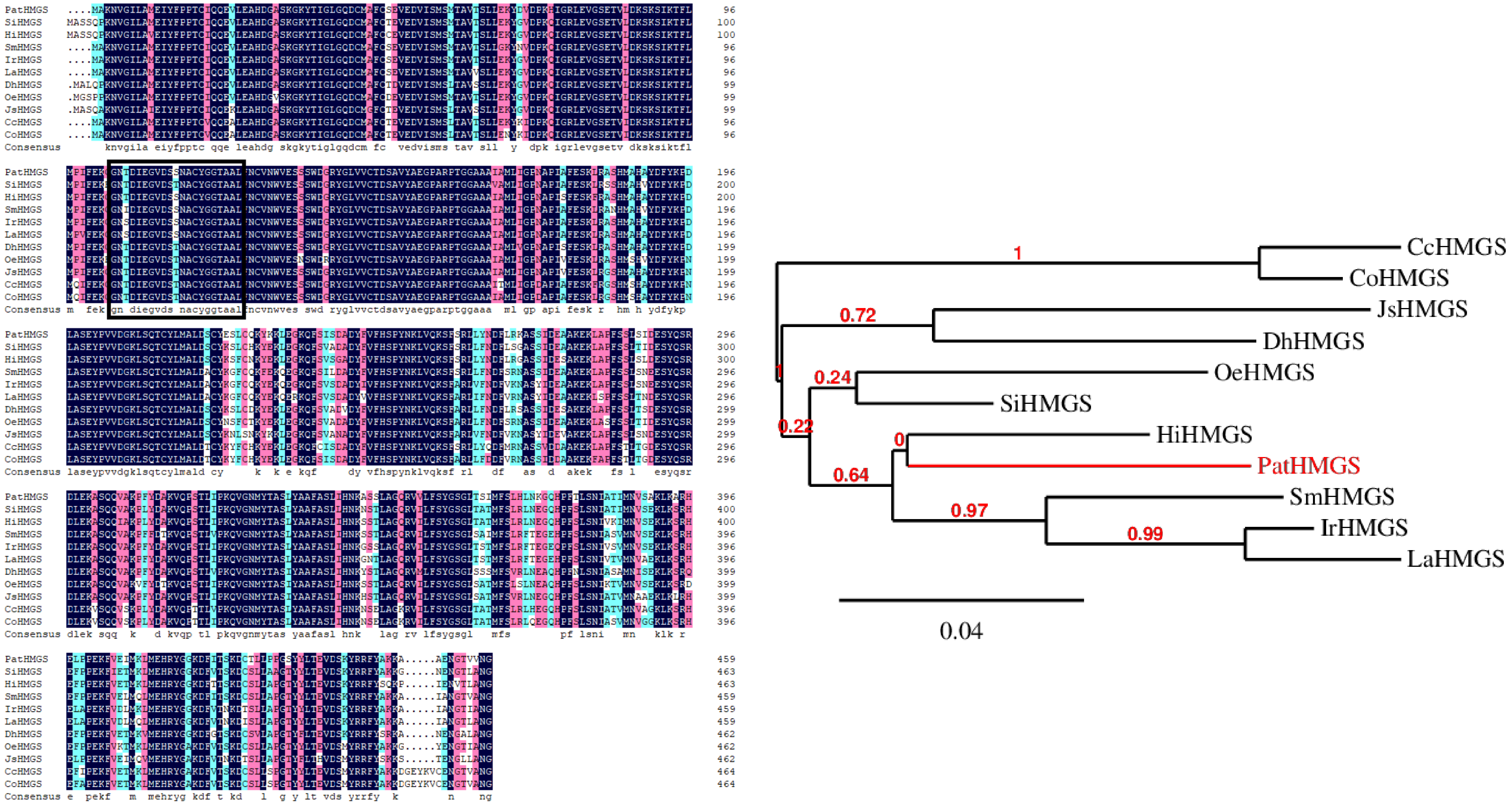

Figure S18. Sequence alignment and phylogenetic relationships of HMGS proteins from *P. cablin* and various other plants. Proteins included are SiHMGS (Sesamum indicum, XM 011082747.2), HiHMGS (Handroanthus impetiginosus, PIN08999.1), SmHMGS (Salvia miltiorrhiza, FJ785326.1), IrHMGS (Isodon rubescens, KX151718.1), LaHMGS (Lavandula angustifolia, JX630154.1), DhHMGS (Dorcoceras hygrometricum, KZV55860.1), OeHMGS (Olea europaea var. sylvestris, XM 023037056.1), JsHMGS (Jasminum sambac, ATE62981.1), CcHMGS (Corchorus capsularis, OMO89642.1), CoHMGS (Corchorus olitorius, OMO81028.1)), and *P. cablin* PatHMGS.

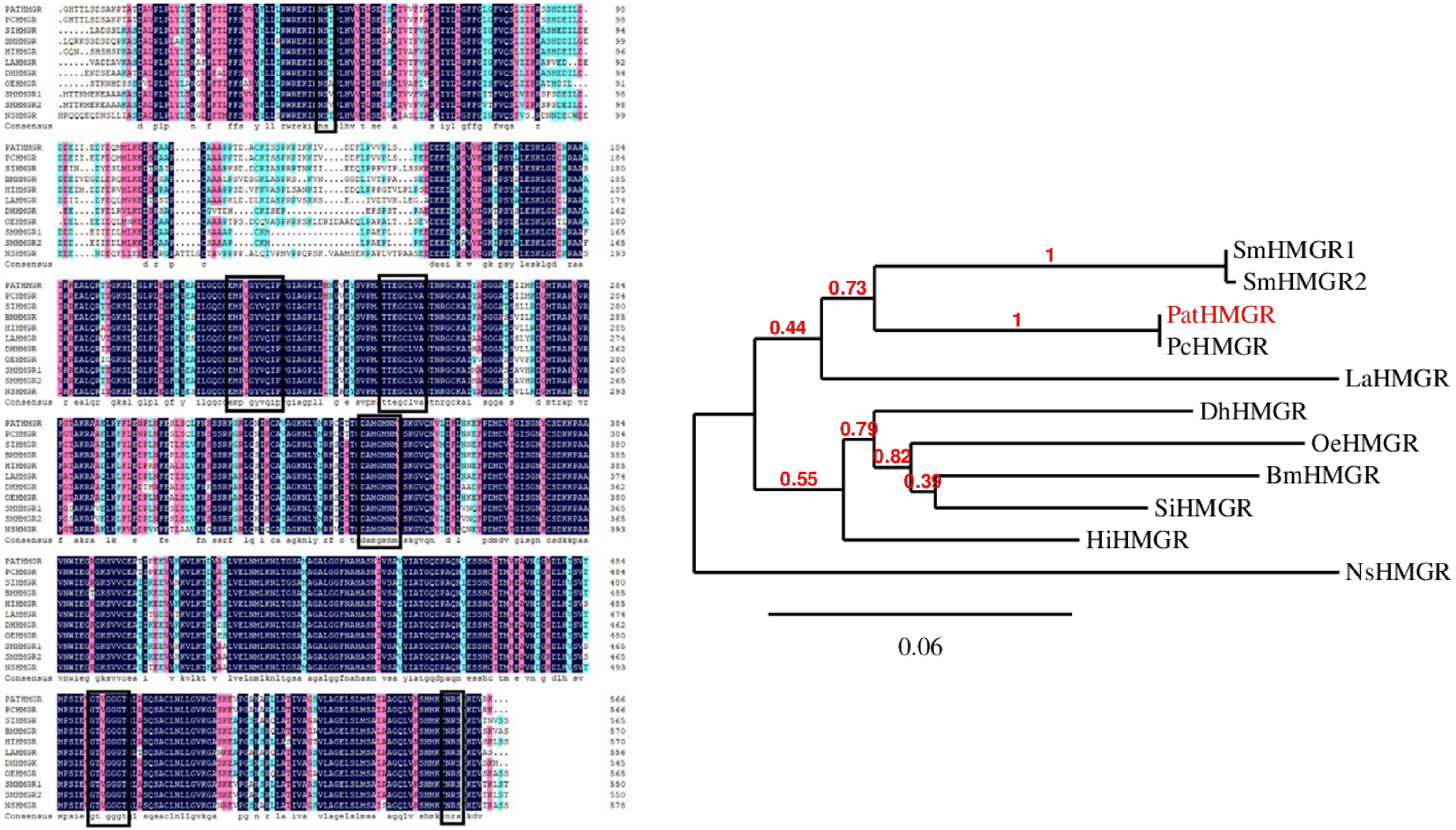

Figure S19. Sequence alignment and phylogenetic relationships of HMGR proteins from *P. cablin* and various other plants. Proteins included are PcHMGR (Pogostemon cablin, KF926079.1), SiHMGR (Sesamum indicum, XP 011092919.1), BmHMGR (Bacopa monnieri, HM222606.1), HiHMGR (Handroanthus impetiginosus, PIN14781.1), LaHMGR (Lavandula angustifolia, AGQ04159.1), DhHMGR (Dorcoceras hygrometricum, KZV41542.1), OeHMGR (Olea europaea var. sylvestris, XM 023040880.1), NsHMGR (Nicotiana sylvestris, NP 001289522.1), SmHMGR1 (Salvia miltiorrhiza, GU367911.1) and SmHMGR2 (Salvia miltiorrhiza,FJ747636.1), and *P. cablin* PatHMGR. The putative HMG-CoA-binding sites (EMPIGYVQIP and TTEGCLVA), NADP(H)-binding sites (DAMGMNM and GTVGGGT) , N-linked glycosylation sites (N-X-S/T) and N-glycosylation site (NST) is

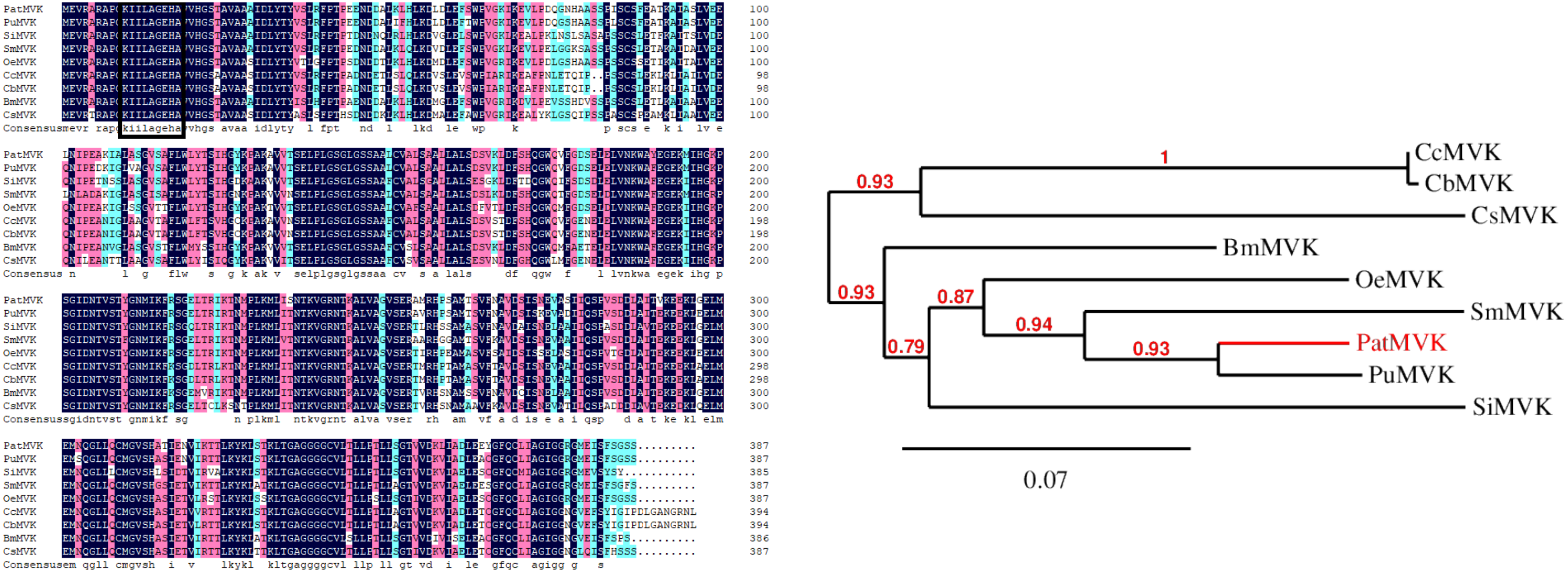

Figure S20. Sequence alignment and phylogenetic relationships of MVK proteins from *P. cablin* and various other plants. Proteins included are PuMVK (Phlomis umbrosa, KU317505.1), SiMVK (Sesamum indicum, XM 011093722.2), SmMVK (Salvia miltiorrhiza, AEZ55674.1), OeMVK (Olea europaea var. sylvestris, XP 022870032.1), BmMVK (Bacopa monnieri, JQ670899.1), CsMVK (Camellia sinensis, MF668187.1)), and *P. cablin* PatMVK. The peroxisomal targeting signal 2 (PTS2) related nonapeptide (KIILAGEHA) is boxed.

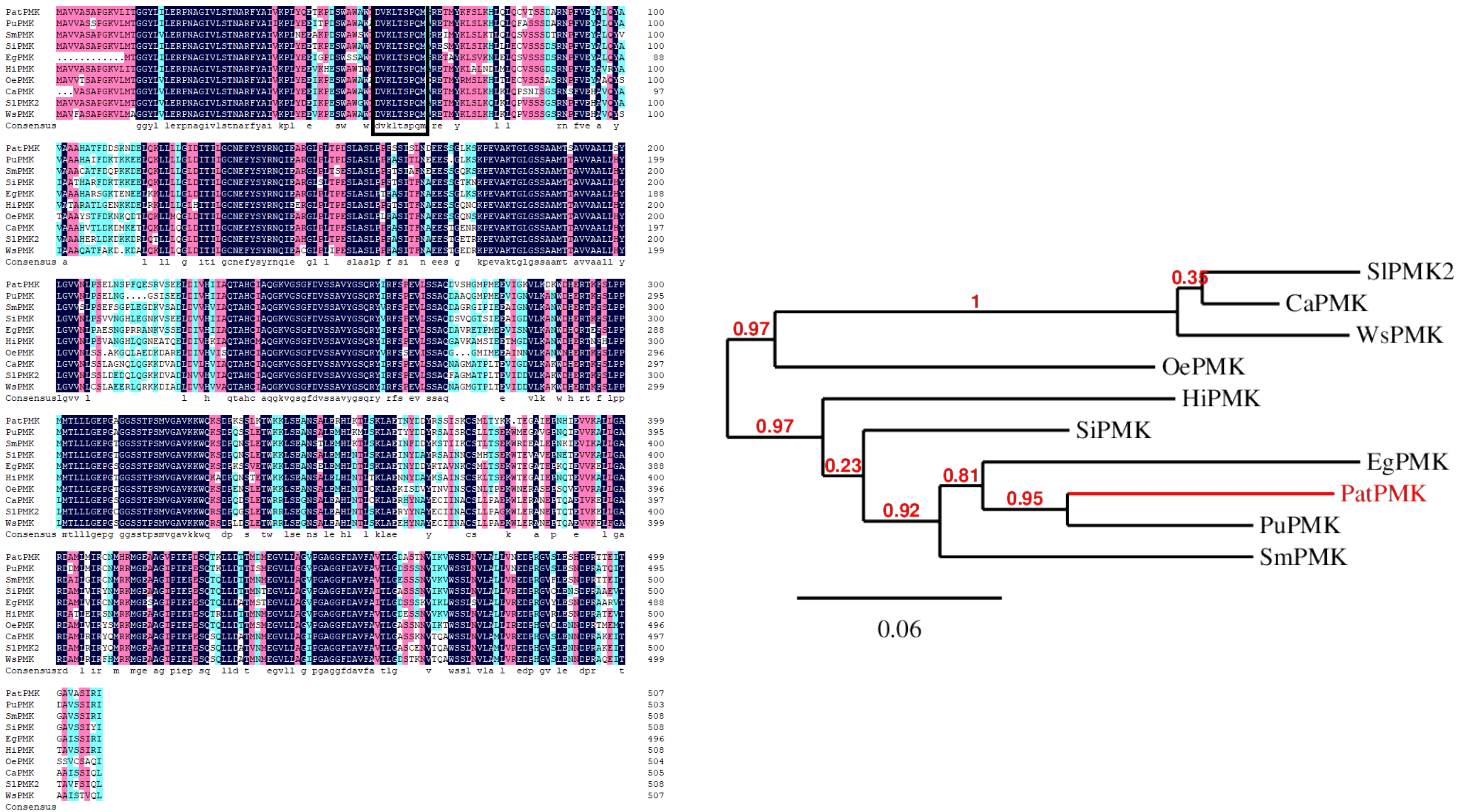

Figure S21. Sequence alignment and phylogenetic relationships of PMK proteins from *P. cablin* and various other plants. PMK proteins included are PuPMK (Phlomis umbrosa, KU317506.1), SmPMK (Salvia miltiorrhiza, JN831095.1), SiPMK (Sesamum indicum, XM 011076340.2), EgPMK (Erythranthe guttata, XM 012991812.1), HiPMK (Handroanthus impetiginosus, PIN17872.1), OePMK (Olea europaea var. sylvestris, XM 023006964.1), CaPMK (Capsicum annuum, XM 016716080.1), SlPMK2 (Solanum lycopersicum, NM 001366055), and *P. cablin* PatPMK. The peroxisomal targeting signal 2 (PTS2) related nonapeptide (DVKLTSPQL/M) is boxed.

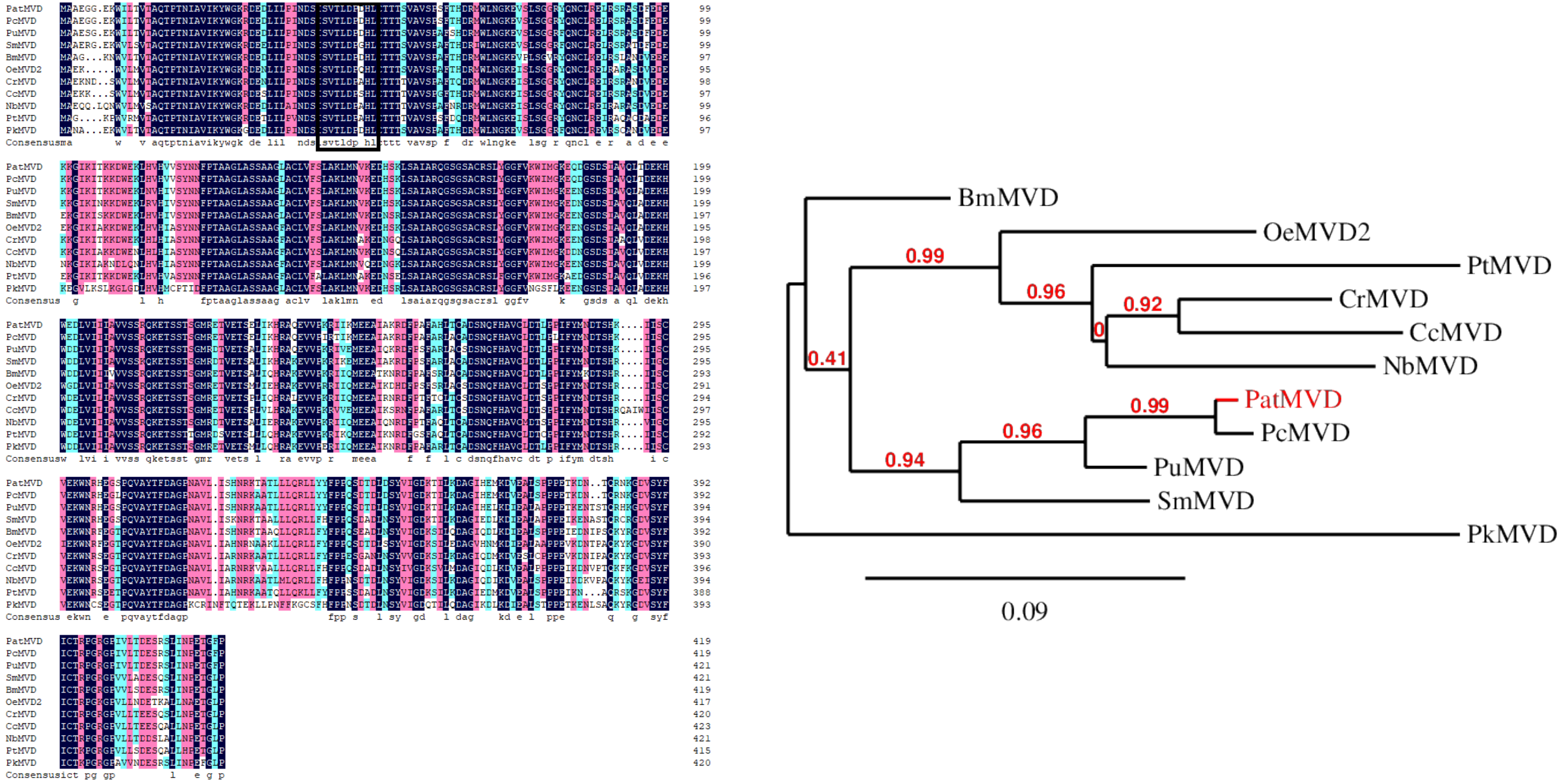

Figure S22. Sequence alignment and phylogenetic relationships of MVD proteins from *P. cablin* and various other plants. MVD proteins included are PcMVD (Pogostemon cablin, KF926083.1), PuMVD (Phlomis umbrosa, KU317504.1), SmMVD (Salvia miltiorrhiza, JN831105.1), BmMVD (Bacopa monnieri, KM896879.1), OeMVD2 (Olea europaea var. sylvestris, XM 023004709.1), Cr MVD (Catharanthus roseus, ADR65113.1), CcMVD (Coffea canephora, CDO99902.1), PtMVD (Populus trichocarpa, XP 002315441.1), PkMVD (Picrorhiza kurrooa, KC958912.1), and *P. cablin* PatMVD. The peroxisomal targeting signal 2 (PTS2) related nonapeptide (SVTLDPXHL) is boxed.

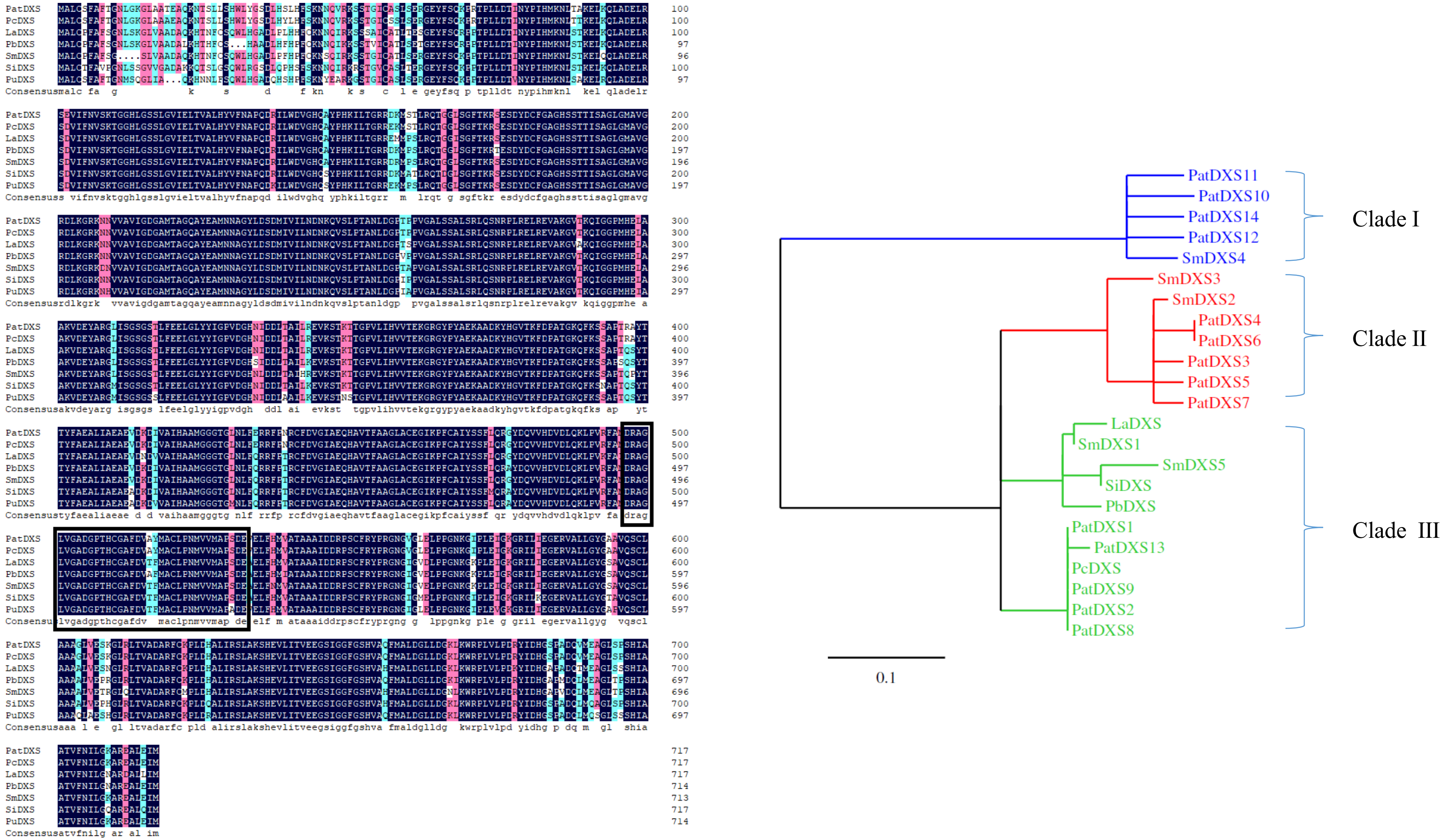

Figure S23. Sequence alignment and phylogenetic relationships of DXS proteins from *P. cablin* and various other plants. DXS proteins included are PcDXS (Pogostemon cablin, KF926078), LaDXS (Lavandula angustifolia, JX630149.1), PbDXS (Plectranthus barbatus, KU178948.1), SmDXS (Salvia miltiorrhiza, EU670744.1), SiDXS (Sesamum indicum, XM 011092570.2), and *P. cablin* PatDXS. The consensus thiamine pyrophosphatase - binding motif (GDGAMTAG……VILND) and the pyridine binding domain (DRAGLVGAD……VMAPSD) is boxed.

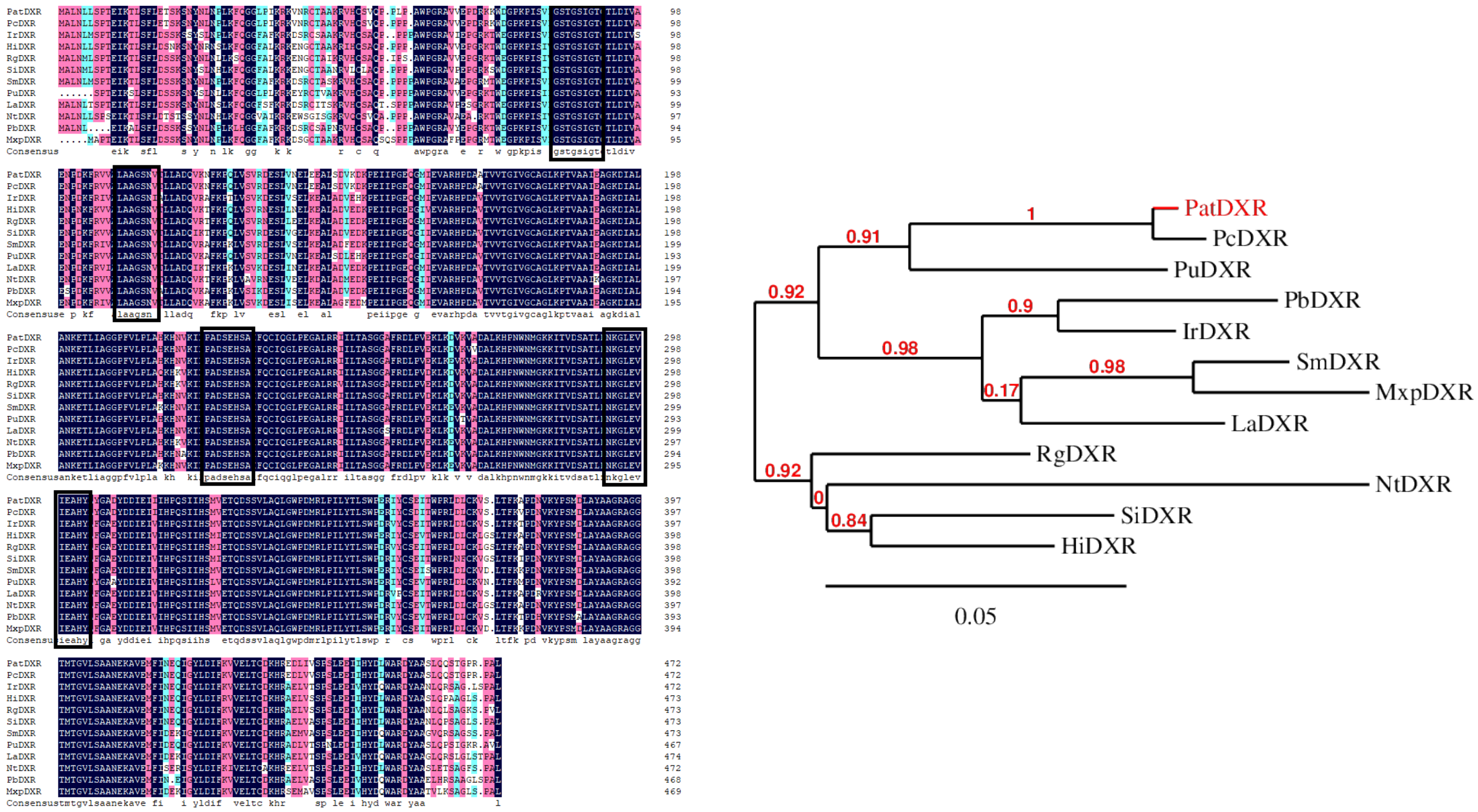

Figure S24. Sequence alignment and phylogenetic relationships of DXR proteins from *P. cablin* and various other plants. DXR proteins included are PcDXR (Pogostemon cablin, KF926086.1), IrDXR (Isodon rubescens, KT948059.1), RgDXR (Rehmannia glutinosa, KX058461.1), SiDXR (Sesamum indicum, XM 011073548.2), SmDXR (Salvia miltiorrhiza, FJ476255.1), PuDXR (Phlomis umbrosa, KU317509.1), LaDXR (Lavandula angustifolia, JX630151.1), PbDXR (Plectranthus barbatus, AY515699.1), HiDXR (Handroanthus impetiginosus, PIN15525.1) and NtDXR (Nicotiana tabacum, NP 001312964.1)), and *P. cablin* PatDXR. The putative two NADPH binding motif (GSTGSIGT and LAAGSNV) and two active sites (PADSEHSA and NKGLEVIEAHY) is boxed.

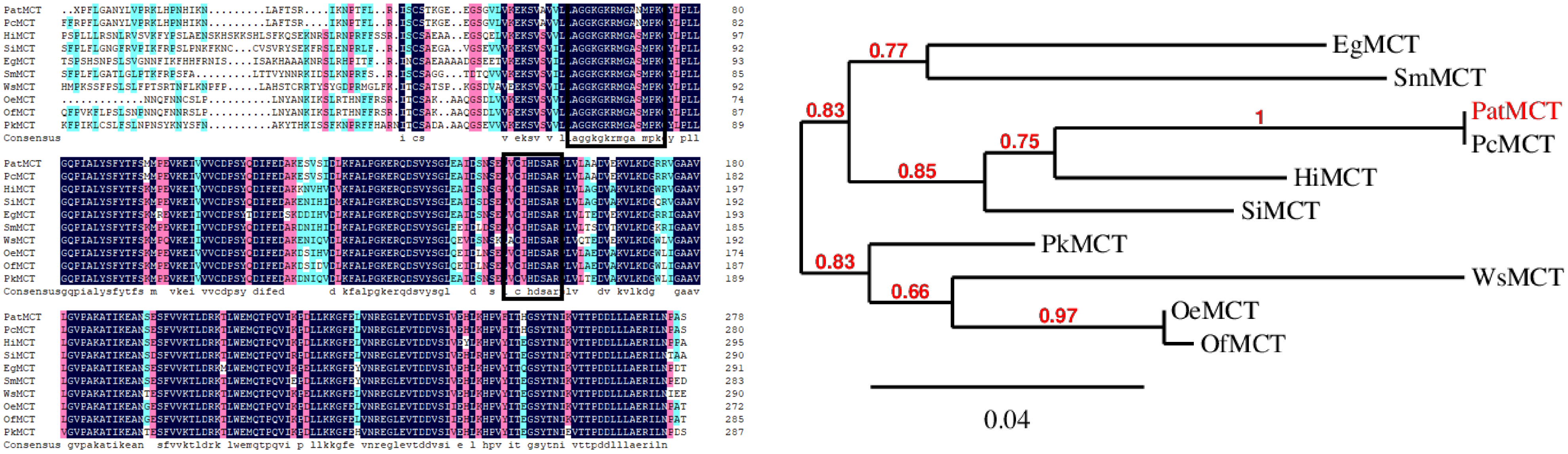

Figure S25. Sequence alignment and phylogenetic relationships of MCT proteins from *P. cablin* and various other plants. MCT proteins included are PcMCT (Pogostemon cablin, KF926080.1), HiMCT (Handroanthus impetiginosus, PIN03655.1), SiMCT (Sesamum indicum, XM 011089302.2), EgMCT (Erythranthe guttata, XM 012986919.1), SmMCT (Salvia miltiorrhiza, AEZ55666.1), OeMCT (Olea europaea, XP 022895626.1), OfMCT (Osmanthus fragrans, AOT86858.1), WsMCT (Withania somnifera, AOX15282.1), PkMCT (Picrorhiza kurrooa, JQ991625), and *P. cablin* PatMCT.

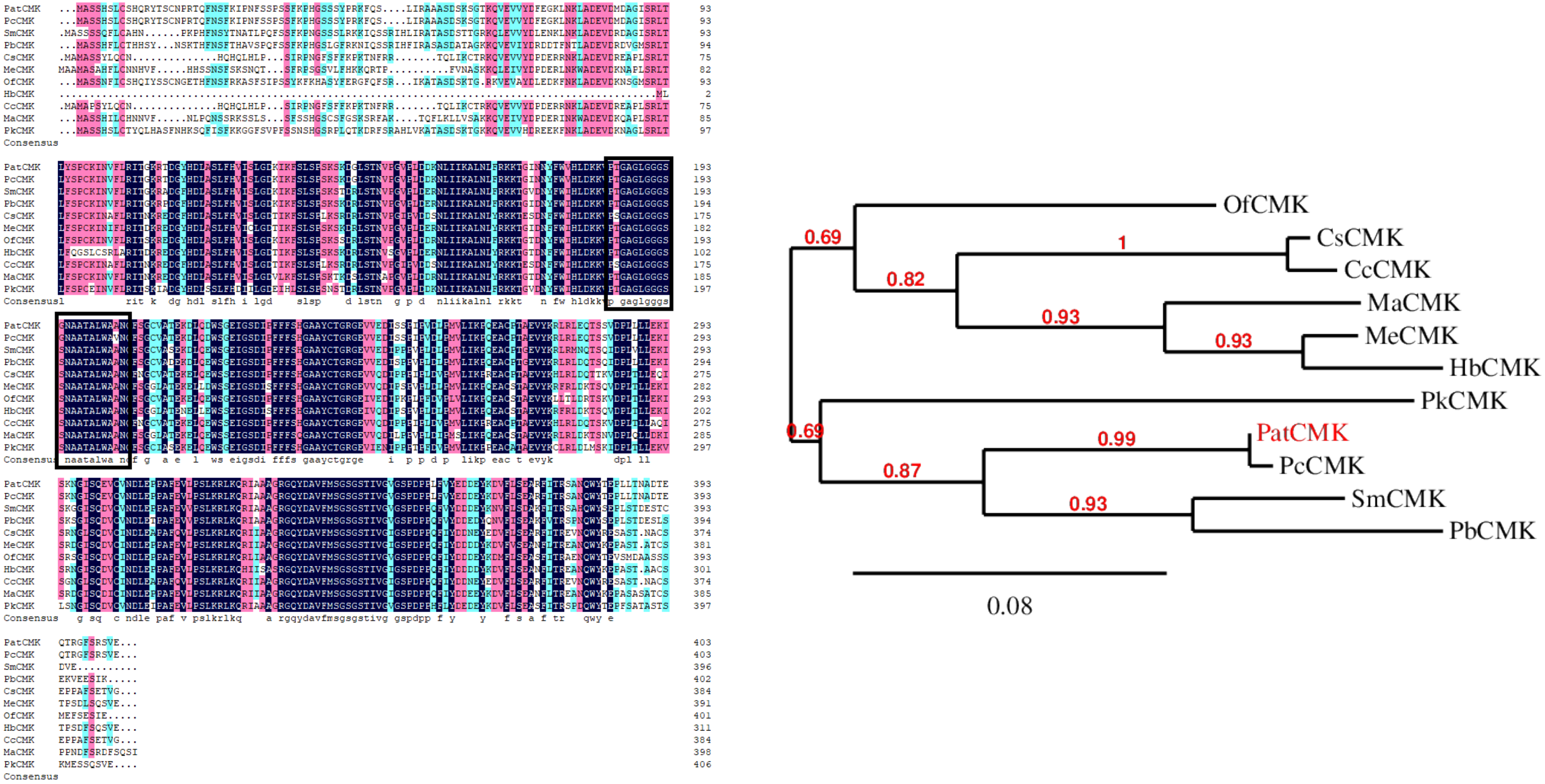

Figure S26 Sequence alignment and phylogenetic relationships of CMKs form P. cablin and various other plants. CMK proteins included are PcCMK (Pogostemon cablin, KF926081.1), SmCMK (Salvia miltiorrhiza, EF534309.1), PbCMK (Plectranthus barbatus, AOZ60046.1), CsCMK (Citrus sinensis ,KDO66318.1), MeCMK (Manihot esculenta, XP 021630383.1), OfCMK (Osmanthus fragrans, AOT86859.1), HbCMK (Hevea brasiliensis,XP 021677484.1), CcCMK (Citrus clementina, XP 006446412.1), MaCMK (Morus alba, AOV62776.1) , PkCMK (Picrorhiza kurrooa, EF199769)), and *P. cablin* PatCMK was used as an out group.

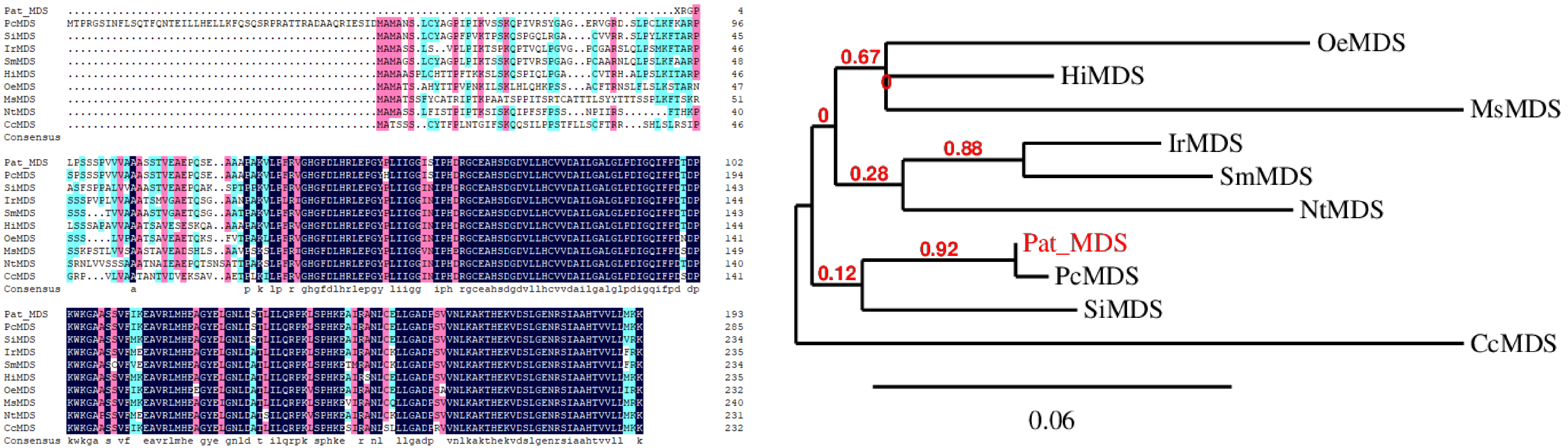

Figure S27. Sequence alignment and phylogenetic relationships of MDSs form P. cablin and various other plants. MDS proteins included are PcMDS (Pogostemon cablin, KF926082.1), SiMDS (Sesamum indicum, XP 011073285.1), IrMDS (Isodon rubescens, KT948057.1), SmMDS (Salvia miltiorrhiza, AEZ55667.1), HiMDS (Handroanthus impetiginosus, PIN08540.1), OeMDS (Olea europaea var. sylvestris, XP 022892962.1), MsMDS (Mitragyna speciosa, AFB70982.1), NtMDS (Nicotiana tabacum, NP 001312116.1), CcMDS (Cynara cardunculus var. Scolymus, XP 024996906.1), and *P. cablin* PatMDS was used as an out group.

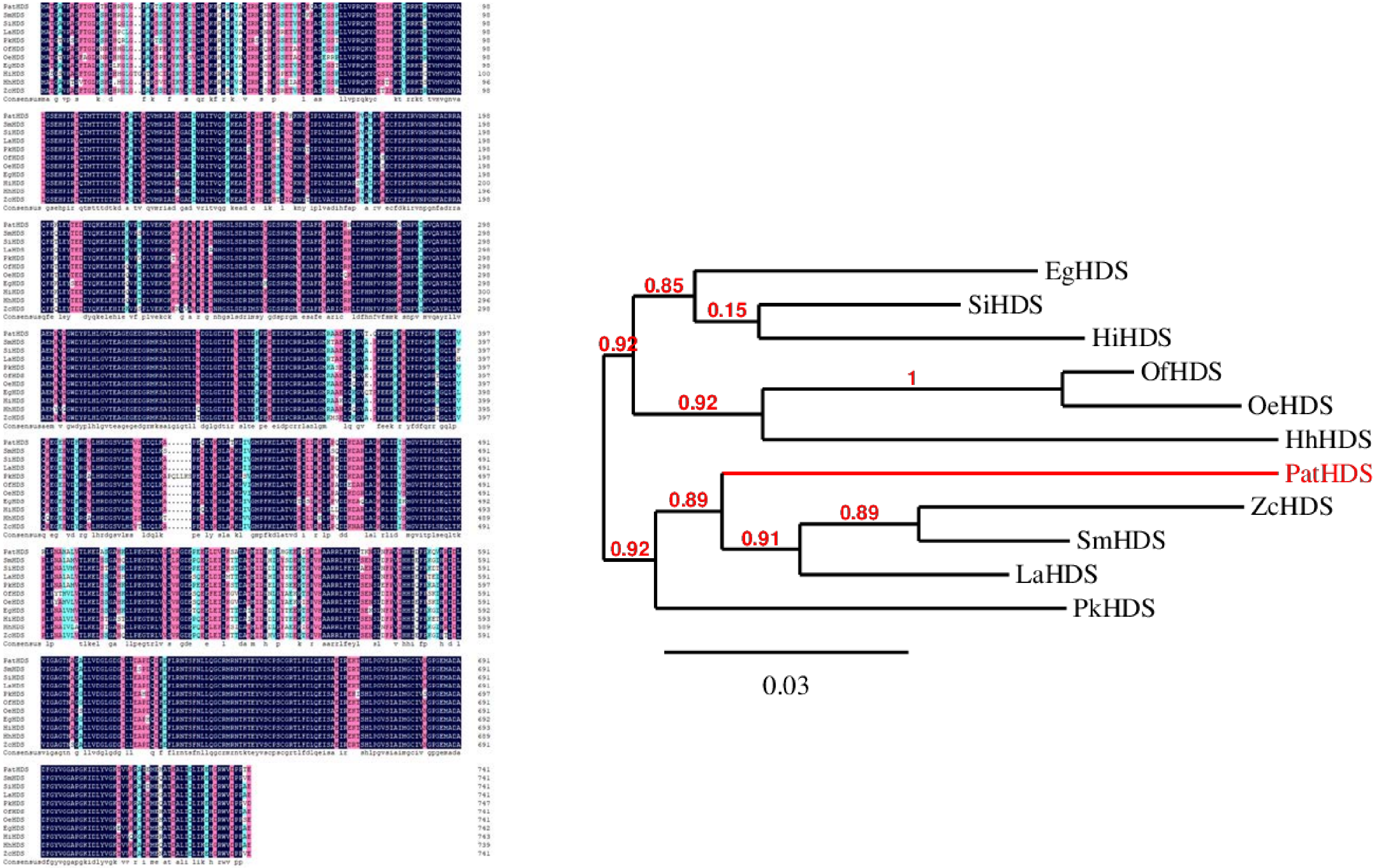

Figure S28. Sequence alignment and phylogenetic relationships of HDSs form P. cablin and various other plants. HDS proteins included are SmHDS (Salvia miltiorrhiza, KJ746807.1), SiHDS (Sesamum indicum, XM 011091597.2), LaHDS (Lavandula angustifolia, JX630152.1), PkHDS (Picrorhiza kurrooa, KF360032.1), OfHDS (Osmanthus fragrans,KX400847.1), OeHDS (Olea europaea var. sylvestris, XM 023032099.1), EgHDS (Erythranthe guttata, XM 012983183.1), HiHDS (Handroanthus impetiginosus, PIN26717.1), HhHDS (Hedera helix, APY22346.1), ZcHDS (Ziziphora clinopodioides, MG065856)), and *P. cablin* PatHDS was used as an out group.

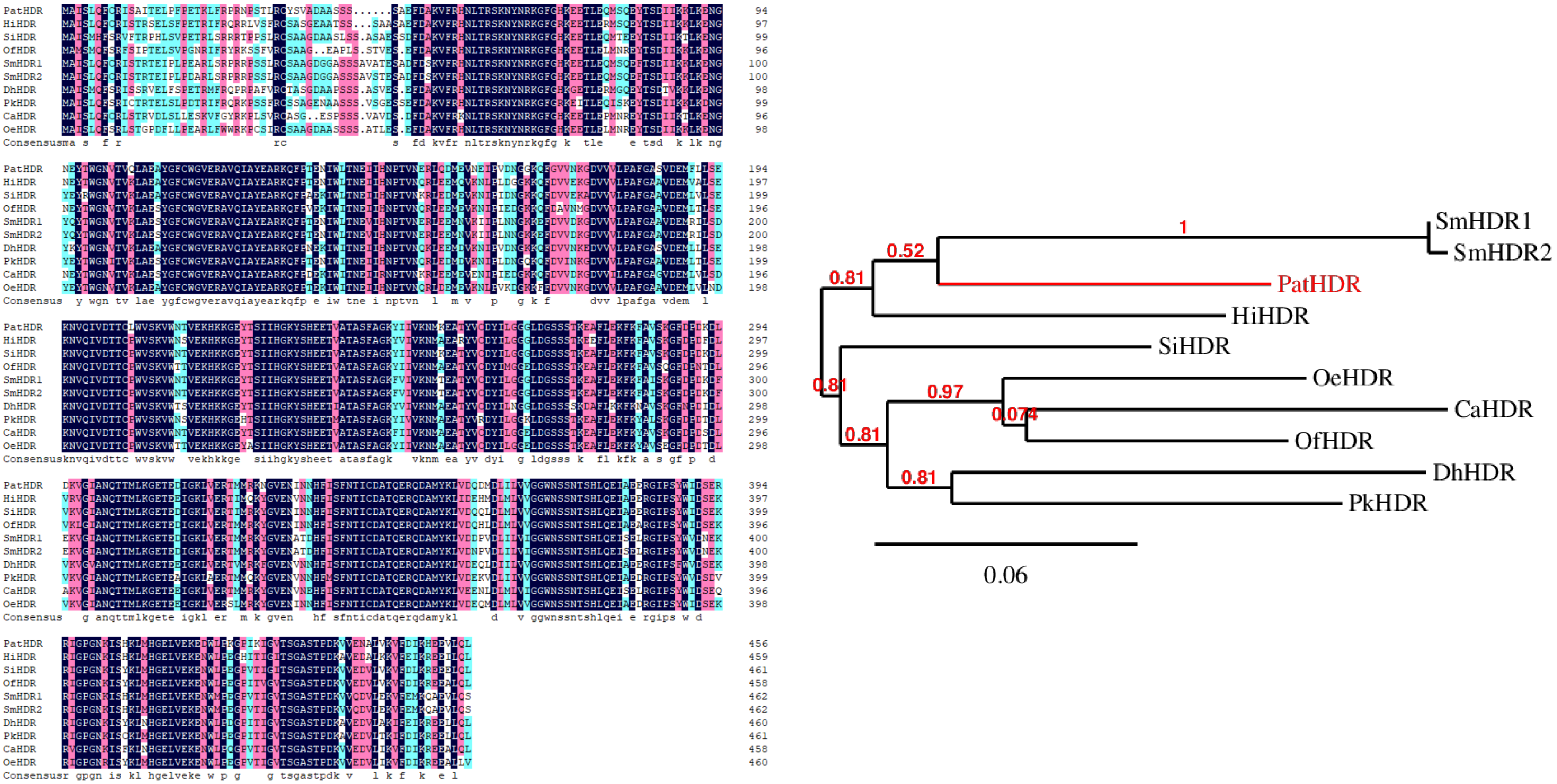

Figure S29. Sequence alignment and phylogenetic relationships of HDRs form *P. cablin* and various other plants. HDR proteins included are HiHDR (Handroanthus impetiginosus, PIN22132.1), SiHDR (Sesamum indicum, XM 011090033.2), OfHDR (Osmanthus fragrans, AOT86863.1), SmHDR1 (Salvia miltiorrhiza, JX233817.1), SmHDR2 (Salvia miltiorrhiza, JX516088.1), DhHDR (Dorcoceras hygrometricum, KZV30833.1), PkHDR (Picrorhiza kurrooa, EF199770.1), CaHDR (Camptotheca acuminate, ABI64152.1), OeHDR (Olea europaea var. sylvestris, XM 023005204.1), and *P. cablin* PatHDR was used as an out group.

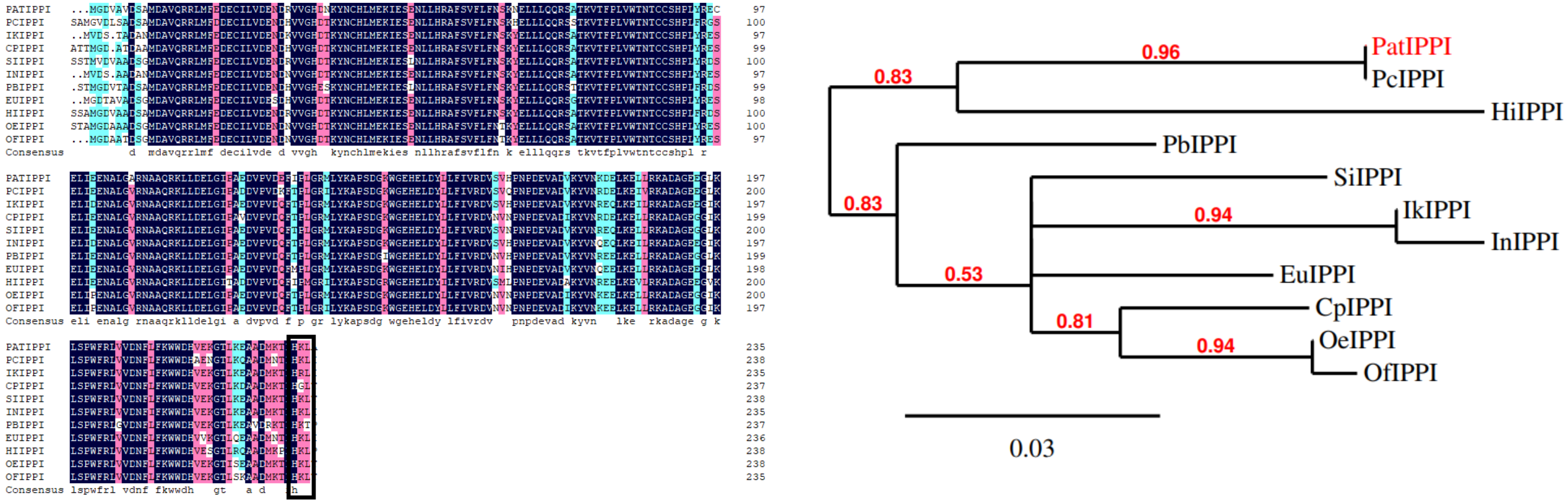

Figure S30. Sequence alignment and phylogenetic relationships of IPPI proteins from *P. cablin* and various other plants. IPPI proteins included are PcIPPI (Pogostemon cablin, KF926084.1), IkIPPI (Ipomoea sp. Kenyan, BAI47570.1), CpIPPI (Carica papaya, XP 021896057.1), SiIPPI (Sesamum indicum, XP 011084658.1), InIPPI (Ipomoea nil, XP 019153180.1), PbIPPI (Plectranthus barbatus, AHG98058.1), EuIPPI (Eucommia ulmoides, AGJ03660.1), HiIPPI (Handroanthus impetiginosus, PIN12895.1), OeIPPI (Olea europaea var. sylvestris, XP 022858957.1), OfIPPI (Osmanthus fragrans, AOT86864.1), and *P. cablin* PatIPPI. The PTS1 related motif (HKL) is boxed.

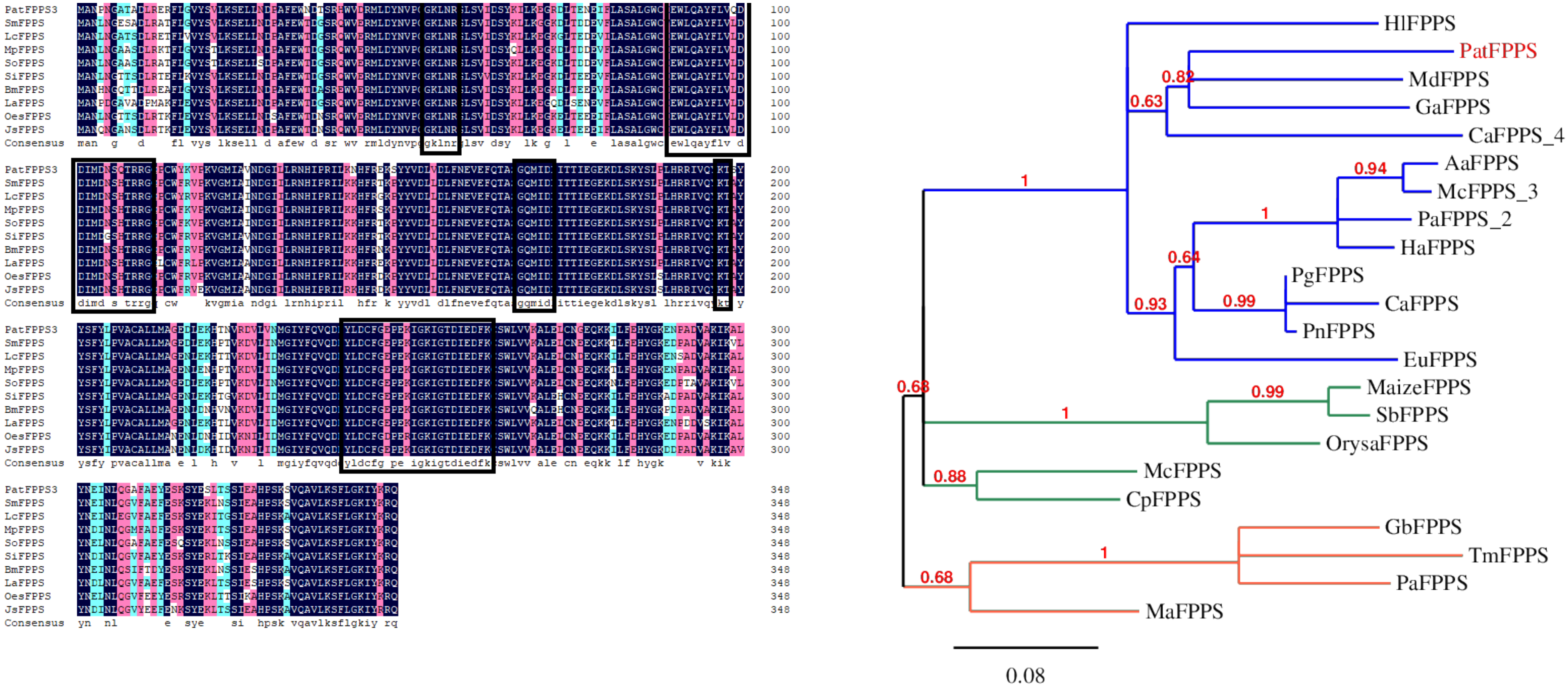

Figure S31. Sequence alignment and phylogenetic relationships of FPPS proteins from *P. cablin* and various other plants. Proteins included are SmFPPS (ABV08819.1, Salvia miltiorrhiza), LcFPPS (ALT07952.1, Leucosceptrum canum), MpFPPS (AAK63847.1, Mentha x piperita), SoFPPS (AQY54371.1, Salvia officinalis), SiFPPS (XP_011095887.1, Sesamum indicum), BmFPPS (ADV03080.1, Bacopa monnieri), LaFPPS (AGQ04160.1, Lavandula angustifolia), OesFPPS (XP_022879277.1, Olea europaea var. sylvestris) and JsFPPS (AIY24422.1, Jasminum sambac)
